# CD56^neg^ CD16^+^ cells represent a distinct mature NK cell subset with altered phenotype and are associated with adverse clinical outcome upon expansion in AML

**DOI:** 10.3389/fimmu.2024.1487792

**Published:** 2025-01-10

**Authors:** Julia Wlosik, Florence Orlanducci, Manon Richaud, Clemence Demerle, Amira Ben Amara, Marie-Sarah Rouviere, Philippe Livrati, Laurent Gorvel, Marie-Anne Hospital, Nicolas Dulphy, Raynier Devillier, Norbert Vey, Daniel Olive, Anne-Sophie Chretien

**Affiliations:** ^1^ Team Immunity and Cancer, Cancer Research Center of Marseille (CRCM), Inserm U1068, CNRS UMR7258, Paoli-Calmettes Institute, University of Aix-Marseille UM105, Marseille, France; ^2^ Immunomonitoring Department, Paoli-Calmettes Institute, Marseille, France; ^3^ Cytometry Platform, Cancer Research Center of Marseille (CRCM), Inserm U1068, CNRS UMR7258, Paoli-Calmettes Institute, University of Aix-Marseille UM105, Marseille, France; ^4^ Centre for Clinical Investigation in Biotherapy, Paoli-Calmettes Institute, University of Aix-Marseille, Inserm CBT 1409, Marseille, France; ^5^ Hematology Department, CRCM, Inserm U1068, CNRS UMR7258, Paoli-Calmettes Institute, Marseille, France; ^6^ Paris Cité University, Saint-Louis Research Institute, Inserm UMRS1160, Paris, France; ^7^ Immunology and Histocompatibility Laboratory, Assistance Publique-Hôpitaux de Paris, Saint-Louis Hospital, Paris, France

**Keywords:** CD56 neg NK cells, AML, high-dimensional cytometry, RNA-sequencing, cancer

## Abstract

**Introduction:**

Acute myeloid leukemia (AML) is a rare haematological cancer with poor 5-years overall survival (OS) and high relapse rate. Leukemic cells are sensitive to Natural Killer (NK) cell mediated killing. However, NK cells are highly impaired in AML, which promote AML immune escape from NK cell immune surveillance. We made the first report of CD56neg CD16+ NK cells expansion in AML. This unconventional subset has been reported to expand in some chronic viral infections. Although it is unclear whether CD56neg NK cells expansion mechanism is common across diseases, it seems more relevant than ever to further investigate this subset, representing a potential therapeutic target.

**Methods:**

We used PBMCs from AML patients and HV to perform mass cytometry, spectral flow cytometry, bulk RNA-seq and in vitro assays in order to better characterize CD56neg CD16+ NK cells that expand in AML.

**Results:**

We confirmed that CD56neg CD16+ NK cells represent a unique NK cell subset coexpressing Eomes and T-bet. CD56neg CD16+ NK cells could recover CD56 expression in vitro where they displayed unaltered NK cell functions. We previously demonstrated that CD56neg CD16+ NK cells expansion at diagnosis was associated with adverse clinical outcome in AML. Here, we validated our findings in a validation cohort of N=38 AML patients. AML patients with CD56neg CD16+ NK cells expansion at diagnosis had decreased overall survival (HR[CI95]=5.5[1.2-24.5], p=0.0251) and relapse-free survival (HR[CI95]=13.1[1.9-87.5], p=0.0079) compared to AML patients without expansion after 36 months follow-up. RNA-seq unveiled that CD56neg CD16+ NK cells were mature circulating NK cells with functional capacities. Upon expansion, CD56neg CD16+ NK cells from AML patients showed altered proteomic phenotype, with increased frequency of terminally mature CD56neg CD16+ NK cells expressing TIGIT along with decreased frequency of Siglec-7+ CD56neg CD16+ NK cells.

**Discussion:**

Taken together, our results suggest that we could harness CD56neg CD16+ NK cells cytotoxic potential in vitro to restore NK cell anti-tumor response in AML patients with CD56neg CD16+ NK cells expansion and improve patients’ prognosis. To conclude, CD56neg CD16+ NK cells represent a relevant target for future NK-cell-based immunotherapies in AML.

## Introduction

NK cells are innate cytotoxic lymphocytes with a pivotal role in the clearance of pathogens and tumor cells (1). They can be divided into three distinct functional subsets based on the surface expressions of CD56 and CD16: CD56^dim^ CD16^+^, CD56^dim^ CD16^-^ and CD56^bright^ NK cells. Recently, a growing interest in CD56^neg^ NK cells has arisen. Indeed, this subset present in healthy volunteers, can significantly increase in pathological context and is thought to be a hallmark of chronic NK cell activation (2). Their expansion was first identified in human immunodeficiency virus-1 (HIV-1) infected patients (3) and further characterized by Mavilio and al (4). Despite no consensus on their maturation stage and functional capacities, there are convincing evidence of their clinical implications in various diseases including HIV-1 (5), hepatitis C virus infection (6), malaria (7) or AML as described in our previous study (8).

AML is a hematological malignancy developing within the bone marrow and characterized by a maturation arrest of myeloid precursors and an uncontrolled clonal expansion of poorly differentiated cells, called leukemic blasts. AML is sensitive to NK cell-mediated cytotoxicity, however, leukemic blasts can implement escape mechanisms, resulting in impaired NK cells and poor prognosis (9, 10). We demonstrated that an expansion of CD56^neg^ NK cells higher than a threshold of 10% at diagnosis was associated with adverse clinical outcome in AML. Interestingly, it persisted in some patients after complete remission.

Although progress has been made in AML therapies over the past decades, it still suffers from poor prognosis in patients aged ≥ 60 with a 5-years survival rate below 15% (11). To restore NK cell cytotoxicity, NK cell-based immunotherapies are currently under investigation (12). The role of CD56 in NK cell cytotoxicity is controversial, however, two recent studies (13, 14) demonstrated its implication in the establishment of immunological synapse, likely to be compromised in CD56^neg^ NK cells. Molecules like NK-cell engagers (15) could help circumvent the problem and induce synapse formation, triggering NK cells cytotoxicity. Although it is unclear whether CD56^neg^ NK cells expansion mechanism is common across diseases, it seems more relevant than ever to further investigate this subset, representing a potential therapeutic target.

Hereinafter, we presented evidence that CD56^neg^ CD16^+^ cells define a distinct NK cell subset. CD56^neg^ CD16^+^ NK cells could recover CD56 expression and exhibit potent NK cell functionalities *in vitro* similarly to CD56^+^ NK cells, in a specific culture system. Besides, this subset expressed transcription factors crucial to the maintenance of NK cell identity. Furthermore, we confirmed in a validation cohort of N=38 AML patients that CD56^neg^ CD16^+^ expansion above 10% of total NK cells at diagnosis is associated with adverse clinical outcome. Next, we established a transcriptomic signature of CD56^neg^ CD16^+^ NK cells characterized by hallmarks of mature circulating cytotoxic NK cells, although some transcripts were indicative of impaired cytotoxicity. Finally, we showed that expanded CD56^neg^ CD16^+^ NK cells exhibit a distinct phenotype characterized by the increased frequency of terminally mature CD56^neg^ CD16^+^ NK cells expressing TIGIT and a decreased frequency of Siglec-7+ CD56^neg^ CD16^+^ NK cells.

## Materials and methods

### Study design and patient enrolment

New data generated in this study were obtained from frozen peripheral blood mononuclear cells (PBMCs) of patients (N=38) enrolled in the HEMATOBIO cohort (NCT02320656). Blood collection, PBMCs extraction, and storage were performed by the Paoli-Calmettes Tumor Bank, which operates under authorization #AC-2007-33 granted by the French Ministry of Research. Patients with newly diagnosed AML, except acute promyelocytic leukemia, were eligible for study participation. All patients had received standard chemotherapy. The cohort was divided according to the frequency of CD56^neg^ CD16^+^ NK cells with a threshold of 10% as previously defined (8). The clinical information of all patients is summarized in **Table 1**. Healthy volunteers (HV, N = 16) were recruited from the French Blood Agency (EFS). Written informed consent was obtained from all patients in accordance with the Declaration of Helsinki. The study was reviewed and approved by the Institutional Review Boards of Paoli-Calmettes Institute.

**Table 1:** Patients' characteristics.

Characteristics	AML	Non-Expanded group	Expanded group
Patients, No. (%)	38 (100)	31 (81.6)	7 (18.4)
Age at diagnosis, mean (SD)	49 (15.6)	49.3(16.1)	47.7 (13.8)
Sex, No. (%)			
Female	23 (60.5)	20 (64.5)	3 (42.9)
Male	15 (39.5)	11 (35.5)	4 (57.1)
ELN, No. (%)			
Favorable	14 (36.8)	13 (41.9)	1 (14.3)
Intermediate	10 (26.3)	8 (25.8)	2 (28.6)
Adverse	6 (15.8)	3 (9.7)	3 (42.8)
NA	8 (21.1)	7 (22.6)	1 (14.3)
Treatment No. (%)			
Chemotherapy	38 (100)	31 (100)	7 (100)
Chemotherapy + Allo-SCT	16 (42.1)	11 (35.5)	5 (71.4)
Treatment response, No. (%)			
CR	27 (71.0)	24 (77.4)	3(42.8)
Relapse	6 (15.8)	4 (12.9)	2 (28.6)
Refractory	5 (13.2)	3 (9.7)	2 (28.6)
Blasts (bone marrow) at diagnosis, mean (SD)	58.6 (26.2)	57.1 (26.8)	65.3 (24.1)
Blasts (blood) at diagnosis, mean (SD)	30.7 (31.0)	32.4 (31.6)	23.1 (29.1)

### Fluorescence-activated cell sorting and RNA extraction

Cryopreserved PBMCs from N=4 AML patients and N=5 age-matched HV were thawed in a 37°C water bath, resuspended in RPMI-1640 (Gibco, Grand Island, NY, USA) supplemented with 10% of heat inactivated fetal bovine serum (FBS) (Gibco), centrifugated for 5 min at 1,500 rpm and incubated in a 37°C, 5% CO2 incubator for 30 min. After resting, cells were filtered on a 30 µm pre-separation filter (Miltenyi), centrifugated and incubated in 1X PBS (Gibco) with the viability marker Live/Dead Fixable Aqua (Thermo Fisher Scientific, Waltham, MA, USA) for 25 min on ice in the dark. After washing in 1X PBS and centrifugation for 5 min at 1,500 rpm twice, cells were incubated with BV605 anti-CD56 (clone NCAM16.2), BV711 anti-CD16 (clone 3G8), BV785 anti-CD45 (cloneHI30), FITC anti-CD13 (clone SJ1D1), FITC anti-CD33 (clone HIM3-4), FITC anti-CD34 (clone 581), PE-CF594 anti-CD3 (clone UCHT1) in Brilliant Stain Buffer (BD Biosciences, Franklin Lakes, NJ, USA) for 30 min on ice in the dark. Before acquisition, cells were passed through a 35 µm cell strainer. Cell sorting of CD56^bright^, CD56^dim^ CD16^-^, CD56^dim^ CD16^+^, and CD56^neg^ CD16^+^ NK cell subsets was performed on a FACS Aria III (BD Biosciences) following the gating strategy outlined in **Supplementary Figure S1**. All used antibodies are listed in **Supplementary Table S1**. After cell sorting, total RNA was immediately isolated with a RNeasy Plus Micro Kit (Qiagen, Hilden, Germany) according to manufacturer’s instructions. High-quality RNA was eluted in 14 µl RNase-free water.

### Bulk RNA-sequencing and data processing

Samples from patients and HV were respectively pooled, at the same total RNA concentration, according to NK cell subtype prior to sequencing. RNA integrity was assessed on a Fragment Analyzer (Agilent, Santa Clara, CA, USA). Library preparation, RNA Capture Sequencing and were performed by IntegraGen (Evry, France). Briefly, libraries were prepared using NEBNext^®^ Ultra™ II Directional RNA Library Prep Kit for Illumina (NEB, Ipswich, MA, USA) according to supplier recommendations. The RNA was capture with the Twist Human Core Exome (Twist Bioscience, San Francisco, CA, USA) + IntegraGen Custom V2 Enrichment Systems. Sequencing was performed on an Illumina NovaSeq platform and 2 × 100 bp paired-end reads were generated. RNA and sequencing quality control metrics are summarized in **Supplementary Table S2**.

Spectral flow cytometry, Cell cloning and functional assays, RNA-sequencing analysis, Mass cytometry analysis and Statistical Analysis can be found in **Supplementary Materials and Methods**.

## Results

### CD3^-^ CD56^neg^ CD16^+^ cells represent a distinct NK cell subset

**Figure 1:**
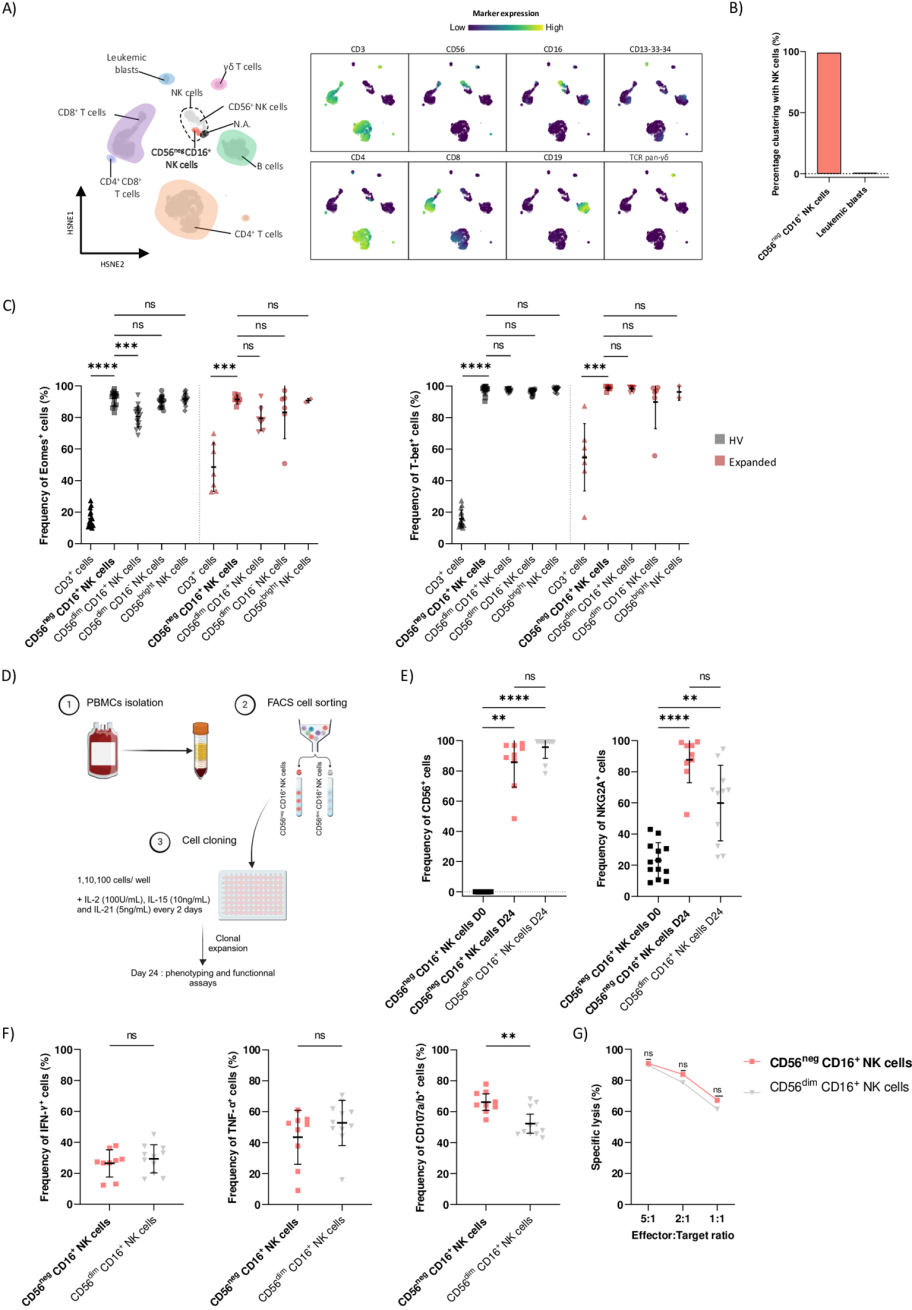
CD3^-^ CD56^neg^ CD16^+^ cells represent a distinct NK cell subset. **(A)** HSNE of 416,000 CD45^+^ cells from peripheral blood of HV and AML patients. Clusters of immune cells are annotated based on the expression of lineage markers. Marker expressions are ArcSinh-5 transformed, blue represents low marker expression and yellow high marker expression. N.A.: not annotated cells. **(B)** Percentage of CD56^neg^ CD16^+^ NK cells and leukemic blasts clustering in NK cells cluster. **(C)** PBMCs from N=16 HV and N=7 AML patients with CD56^neg^ CD16^+^ expansion (Expanded group) were phenotyped using spectral flow cytometry. Boxplots showing frequencies of Eomes^+^ and T-bet^+^ cells among CD3^+^ cells, CD56^neg^ CD16^+^, CD56^dim^ CD16^+^, CD56^dim^ CD16^-^ and CD56^bright^ NK cells were assessed by manual gating. **(D)** Schematic overview of CD56^neg^ CD16^+^ NK cells cloning experiment. Briefly, whole blood from HV was used to isolate PBMCs using density gradient centrifugation. CD56^neg^ CD16^+^ and CD56^dim^ CD16^+^ NK cells were then FACS sorted from collected PBMCs and plated in a 96-wells plate at a concentration of 1, 10 or 100 cells per well. Phenotyping, cytokine production and cytotoxic assays were performed after expansion on day 24. See Material and Methods section for more details. **(E)** Boxplots showing frequencies of CD56^+^ (left) and NKG2A^+^ (right) cells were assessed on CD56^neg^ CD16^+^ and CD56^dim^ CD16^+^ NK cells on days 0 and 24 of culture. **(F)** On day 24, CD56^neg^ CD16^+^ and CD56^dim^ CD16^+^ NK cells were harvested and cocultured with K562 cells at an E:T ratio of 1:1 for 4h at 37°C. Production of IFN-γ and TNF-α and degranulation capacity by CD56^neg^ CD16^+^ and CD56^dim^ CD16^+^ NK cells were assessed. **(G)** Specific lysis by CD56^neg^ CD16^+^ and CD56^dim^ CD16^+^ NK cells against K562 cells was assessed. Statistical significance was determined by Kruskal-Wallis and Dunn *post-hoc* test or Mann-Whitney test. P-values below 0.05 were considered significant. **p<0.01, ***p<0.0001, ****p<0.0001, ns=p not significant. Data is plotted as mean with SD.

Our team made the first description of CD56^neg^ CD16^+^ NK cells expansion in AML patients (8). Therefore, we sought to confirm that CD3^-^ CD56^neg^ CD16^+^ cells identified through mass cytometry belong to NK cell. To this end, we ran Cytosplore^+HSNE^ (16) on our derivation cohort data (8) to perform automatic clustering. We embedded 872 796 CD45^+^ cells following the gating strategy outlined in **Supplementary Figure S2** and isolated from peripheral blood of N=10 HV and N=22 AML patients. Cytosplore^+HSNE^ identified 6 distinct clusters among which NK cells and myeloid/leukemic blasts clusters. Importantly, 88% of CD3^-^ CD56^neg^ CD16^+^ NK cells clustered among NK cell cluster while 12% clustered among leukemic blasts cluster indicating a strong similarity between CD56^neg^ CD16^+^ NK cells and conventional NK cells, i.e. CD56^dim^ CD16^+^,CD56^dim^ CD16^-^ and CD56^bright^ NK cells (p<0.0001) (**Figures 1A, B**).

Eomes and T-bet transcription factors are crucial to sustain NK cell identity and function (17). Using spectral flow cytometry, we assessed the frequencies of Eomes^+^ and T-bet^+^ cells among CD3^+^ cells, CD56^neg^ CD16^+^ and conventional NK cells from PBMCs of HV (N=16) and AML patients (N=7) with CD56^neg^ CD16^+^ NK cells expansion at diagnosis (Expanded group). In the HV group, we observed similar frequency of Eomes^+^ cells among CD56^neg^ CD16^+^, CD56^dim^ CD16^-^ and CD56^bright^ NK cells whereas CD3^+^ cells and CD56^dim^ CD16^+^ NK cells showed lower expression of Eomes compared to CD56^neg^ CD16^+^, respectively p<0.0001 and p=0.0007. In the Expanded group, frequency of Eomes^+^ CD56^neg^ CD16^+^ cells did not differ from the frequencies of conventional NK cells and was significantly increased compared to CD3^+^ cells, p=0.0004 (**Figure 1C**, left panel). Besides, frequencies of T-bet^+^ CD56^neg^ CD16^+^ NK cells from both HV and Expanded groups matched the frequencies of conventional NK cells and were significantly increased compared to CD3^+^ cells, respectively p<0.0001 and p=0.0007 (**Figure 1C**, right panel).

Next, we investigated whether CD56^neg^ CD16^+^ NK cells express NK cell markers and maintained NK cell killing abilities *in vitro*. We performed limiting dilution NK cell cloning from whole blood of HV. PBMCs were isolated using density gradient centrifugation and CD56^neg^ CD16^+^ and CD56^dim^ CD16^+^ NK cells were FACS-sorted before being plated at a concentration of 1, 10 or 100 cells/well in RPMI supplemented with 10% of heat inactivated FBS. Irradiated K562 cells were used as feeders. IL-2 (100U/mL), IL-15 (10ng/mL) and IL-21 (5ng/mL) were added every two days and medium was changed. After 24 days of culture, cells were harvested, phenotyped and functionality was assessed (**Figure 1D**). We observed that expression levels of CD56 and NKG2A were significantly increased in CD56^neg^ CD16^+^ NK cells after 24 days of culture, respectively p=0.0024 and p=0.0003. Importantly, CD56^neg^ CD16^+^ NK cells and CD56^dim^ CD16^+^ NK cells expressed similar levels of CD56 and NKG2A after 24 days in culture (**Figure 1E**). In addition, CD56^neg^ CD16^+^ and CD56^dim^ CD16^+^ NK cells exhibited similar production capacities of IFN-γ and TNF-α after 24 days of culture (**Figure 1F**, left and middle panels). Finally, CD56^neg^ CD16^+^ NK cells expressed higher levels of CD107a/b, p=0.0057, (**Figure 1F**, right panel) and showed similar specific lysis against K562 cells than CD56^dim^CD16^+^ NK cells after 24 days of culture (**Figure 1G**). Together these data support that CD3^-^ CD56^neg^ CD16^+^ cells represent a distinct NK cell subset able to recover CD56 expression *in vitro* where they display unaltered NK cell functions.

### Validation cohort confirmed that CD56^neg^ CD16^+^ NK cells expansion at diagnosis is associated with adverse clinical outcome in AML patients

**Figure 2:**
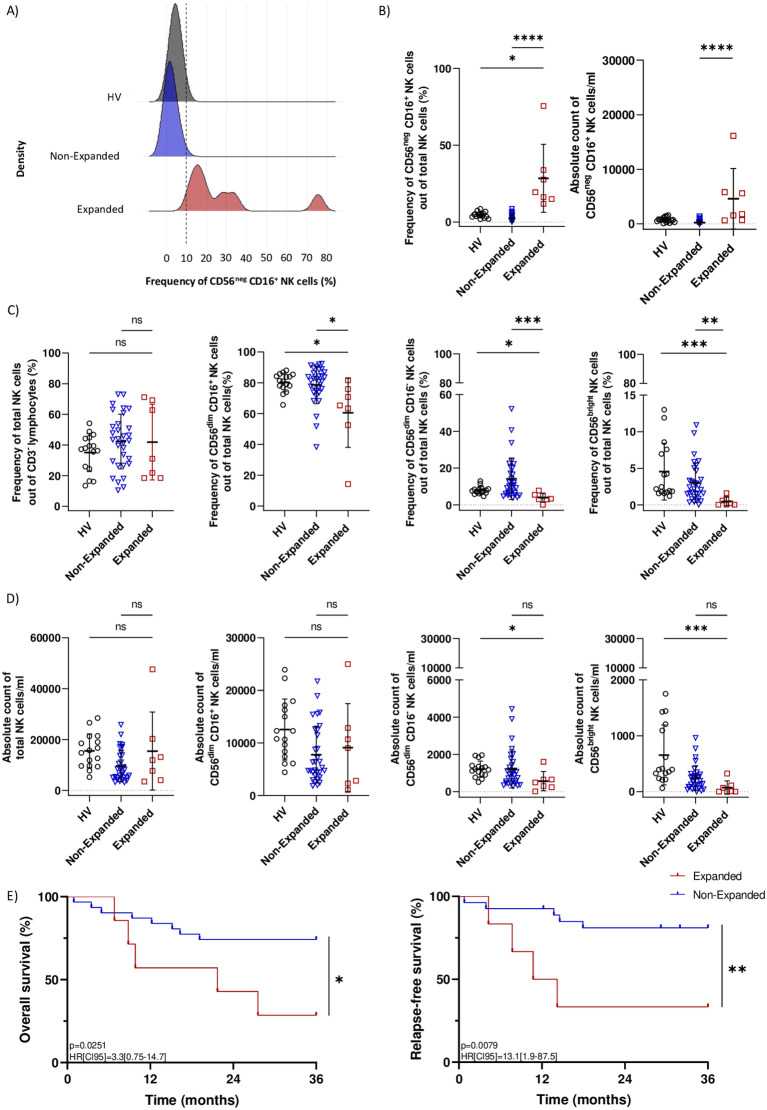
Validation cohort confirmed that CD56^neg^ CD16^+^ NK cells expansion is associated with adverse clinical outcome in AML patients. **(A)** Kernel density estimate of CD56^neg^ CD16^+^ NK cells frequency in N=16 HV, N=31 patients from the Non-Expanded group and N=7 patients from the Expanded group. The dotted line indicates the previously defined 10% threshold used to differentiate Non-Expanded from Expanded patients. **(B)** Boxplot showing the frequency and absolute count of CD56^neg^ CD16^+^ NK cells in HV, Non-Expanded and Expanded patients. **(C)** Boxplots showing frequencies of NK cells, CD56^dim^ CD16^+^ NK cells, CD56^dim^ CD16^-^ NK cells and CD56^bright^ NK cells in HV, Non-Expanded and Expanded patients. **(D)** Boxplots showing absolute counts of NK cells, CD56^dim^ CD16^+^ NK cells, CD56^dim^ CD16^-^ NK cells and CD56^bright^ NK cells in HV, Non-Expanded and Expanded patients. **(E)** Kaplan-Meier curves for overall survival and relapse-free survival of Non-Expanded and Expanded patients. Statistical significance was determined by Kruskal-Wallis and Dunn *post-hoc* test and log-rank test. P-values below 0.05 were considered significant. *p<0.05, **p<0.01, ***p<0.0001, ****p<0.0001, ns, p not significant. Data is plotted as mean with SD.

We demonstrated that CD56^neg^ CD16^+^ NK cells expansion at diagnosis was associated with adverse clinical outcome in AML (8). To validate our previous results, we included N=16 HV, N=31 AML patients without CD56^neg^CD16^+^ NK cells expansion at diagnosis (Non-Expanded group) and N=7 patients in the Expanded group within our validation cohort. Patients’ characteristics are summarized in **Table 1**. PBMCs at diagnosis were analyzed using spectral flow cytometry and NK cells subsets were gated following the gating strategy outlined in **Supplementary Figure S3**. We previously defined a threshold of 10% of CD56^neg^ CD16^+^ NK cells among total NK cells to discriminate between patients. As expected, the threshold could optimally classify samples from the validation cohort in the Non-Expanded or Expanded groups (**Figure 2A**). Indeed, the Expanded group displayed significant CD56^neg^ CD16^+^ NK cells expansion compared to the HV and Non-Expanded groups, respectively p=0.0292 and p<0.001 (**Figure 2B**, left panel). Frequencies of CD56^neg^ CD16^+^ NK cells in the Expanded group ranged from 11.8% to 75.6% (**Supplementary Table S3**). Notably, the Expanded group did not show any increase of total NK cells (**Figure 2C**, left panel). Besides, the Expanded group had significantly less CD56^dim^ CD16^+^, CD56^dim^ CD16^-^ and CD56^bright^ NK cells than the Non-Expanded (p=0.0150, p=0.0007 and p=0.0074 respectively) and HV (p=0.0237, p=0.0432, p=0.0005 respectively) groups (**Figure 2C**). Importantly, absolute count of CD56^neg^ CD16^+^ NK cells was significantly increased in the Expanded group compared to the Non-Expanded group (p<0.0001) (**Figure 2B**, right panel). Besides, absolute counts of total NK cells and CD56^dim^ CD16^+^ NK cells were not altered (Figure D, left panel) while absolute counts of CD56^bright^ and CD56^dim^ CD16^-^ NK cells were decreased in the Expanded group compared to the HV group (Figure D, right panel). However, the paucity of these two subsets supports the hypothesis of an expansion from the CD56^neg^ CD16^+^ NK cells rather than a decrease of the other subsets. On the other hand, we investigated the T cell compartment in the HV, Non-Expanded and Expanded groups and found no difference in the frequencies of CD8^+^ T cells, CD4^+^ T cells, TCRVδ2^+^ T cells and regulatory T cells between groups (**Supplementary Figure S4**).

Among patients in the Expanded group, 42.8% achieved sustained complete remission after first induction therapy versus 77.4% in the Non-Expanded group. Besides, 28.6% of the Expanded group relapsed after first complete remission versus 12.9% in the Non-Expanded group. Importantly, patients from the Expanded group had significantly poorer overall survival (HR[CI95]=3.3[0.75-14.7], p=0.0251) and relapse-free survival (HR[CI95]=13.1[1.9-87.5], p=0.0079) after 36 months follow-up (**Figures 2D, E**). Results from the validation cohort were in line with our previous study (8) and demonstrated that CD56^neg^ CD16^+^ NK cells expansion at diagnosis results in adverse clinical outcome in AML patients.

### CD56^neg^ CD16^+^ NK cells display a unique transcriptomic profile of mature circulating NK cells and show altered expression of transcripts involved in NK cell function

Considering the clinical implications of CD56^neg^ CD16^+^ NK cells expansion in AML, we aimed to further characterize this subset. To this end, we performed bulk RNA-seq profiling of CD56^neg^ CD16^+^, CD56^dim^ CD16^+^, CD56^dim^ CD16^-^ and CD56^bright^ NK cells isolated from peripheral blood in N=5 HV and N=4 AML patients at diagnosis. NK cell subsets from HV and AML patients were respectively pooled prior to analysis. The purity of CD56neg CD16+ NK cells in our dataset was assessed based on cell type-specific transcriptomic signatures for myeloid DC, non-classical monocytes, B cells and T cells adapted from the human protein atlas (18) (https://www.proteinatlas.org/) and low-density neutrophils signature adapted from (19). We set our minimal abundance threshold log10(TPM+1) to 1 to remove the background noise, as suggested in (20). Sorted CD56neg CD16+ NK cells did not express 10/13 and poorly expressed 3/13 transcripts from the non-classical monocytes signature, and did not express transcripts identifying myeloid DC, B cells, T cells or low-density neutrophils (**Supplementary Figure B**). In light of these results, we considered that the purity of CD56neg CD16+ NK cells was satisfactory for downstream analysis. CD56^neg^ CD16^+^ NK cells from HV and AML patients expressed a unique transcriptomic profile with 177 down-regulated and 161 up-regulated transcripts in AML samples and 87 down-regulated and 252 up-regulated transcripts in HV samples (**Supplementary Tables S4**, **S5**). As expected, *NCAM1*, encoding for CD56, was down-regulated in CD56^neg^ CD16^+^ NK cells from both AML patients and HV.

**Figure 3:**
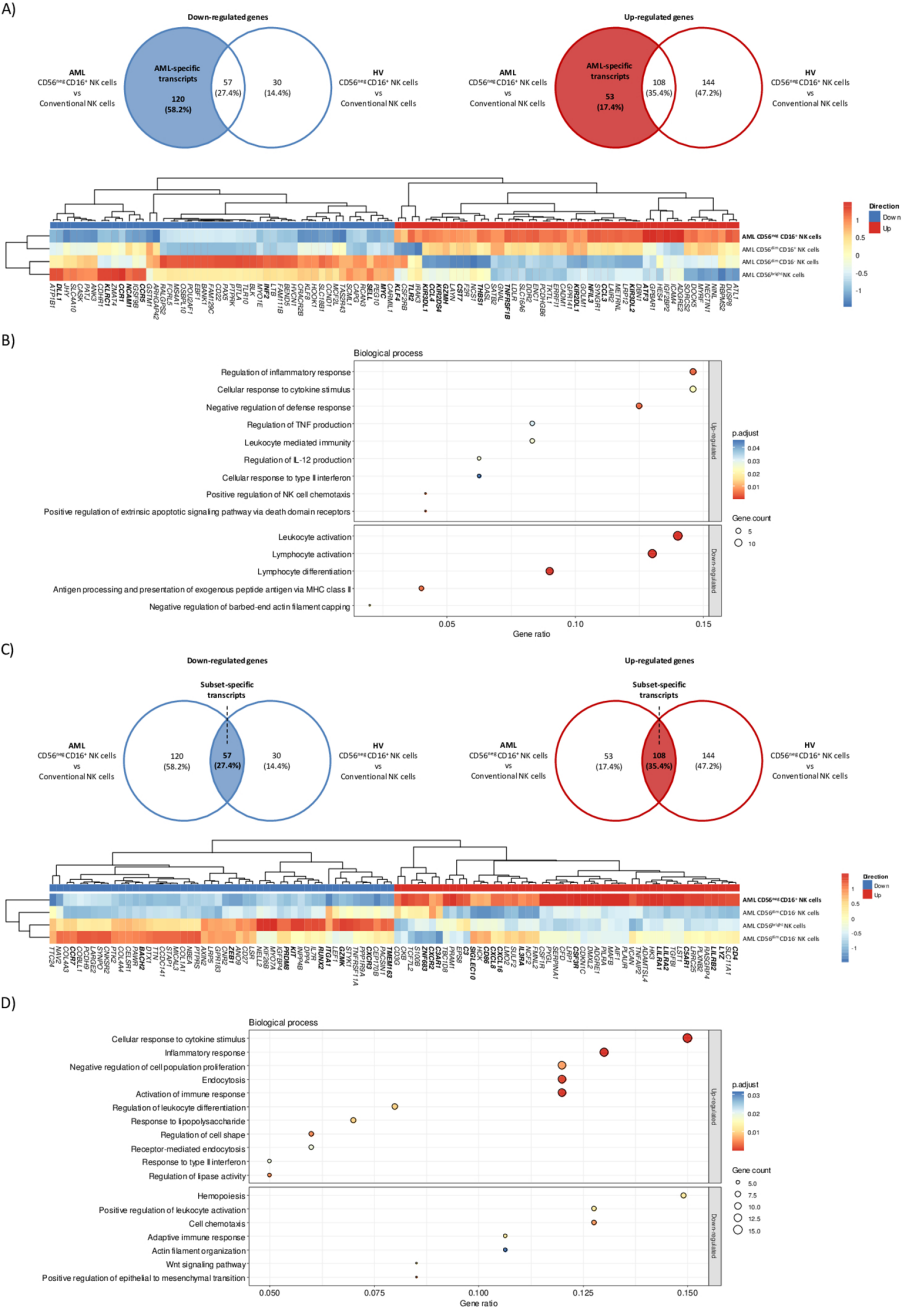
CD56^neg^ CD16^+^ NK cells transcriptomic signature in AML patients. Differential gene expression analysis was performed between CD56^neg^ CD16^+^ *versus* CD56^dim^ CD16^+^, CD56^dim^ CD16 and CD56^bright^ NK cells in HV and AML patients. **(A)** Venn diagrams showing down-regulated (left) and up-regulated (right) AML-specific transcripts in CD56^neg^ CD16^+^ NK cells. Hierarchical clustered heatmap of the top 100 most up and down-regulated AML-specific transcripts in CD56^neg^ CD16^+^ NK cells. Expression is shown as z-score, lowest z-scores are blue and higher z-scores are red. **(B)** Gene ontology enrichment analysis of biological processes for up-regulated (top) and down-regulated (bottom) AML-specific transcripts in CD56^neg^ CD16^+^ NK cells. **(C)** Venn diagrams showing down-regulated (left) and up-regulated (right) subset-specific transcripts in CD56^neg^ CD16^+^ NK cells. Hierarchical clustered heatmap of the top 100 most up and down-regulated subset-specific transcripts in CD56^neg^ CD16^+^ NK cells. Expression is shown as z-scores, lowest z-scores are blue and higher z-scores are red. **(D)** Pathway enrichment analysis of shared up-regulated (top) and down-regulated (bottom) subset-specific transcripts in CD56^neg^ CD16^+^ NK cells. DEG threshold: log2FC>2, average expression>1. Enrichment threshold: adjusted p.value < 0.05.

To assess the impact of AML on CD56^neg^ CD16^+^ NK cells transcriptomic profile, we focused on differentially expressed transcripts unique to the AML group. Venn diagrams identified 120 down-regulated and 53 up-regulated AML-specific transcripts (**Figure 3A**, top panel). We first investigated the maturation status of CD56^neg^ CD16^+^ NK cells from AML patients. This subset down-regulated *MYC*, involved in NK cell development (21) but up-regulated *NFIL3*, an essential nuclear factor for NK cell maturation (22). During maturation, NK cells lose CD62L (*SELL*) and NKG2A (*KLRC1*) expression and acquire KIRs (23). Consistently, CD56^neg^ CD16^+^ NK cells down-regulated *SELL* and *KLRC1* and up-regulated *KIR2DL1*, *KIR3DL1*, *KIR3DL2* and *KIR2DS4*. Besides, *DLL1*, which inhibition is involved in Notch-mediated KIRs expression (24), was also down-regulated. Therefore, CD56^neg^ CD16^+^ NK cells from AML patients seemed to have reached later maturation stage. CD56^neg^ CD16^+^ NK cells expansion could be a consequence of impaired chemokine or cytokine signaling. We observed that CD56^neg^ CD16^+^ NK cells from AML patients down-regulated *CCR1* and *CCR5*, involved in NK cell recruitment in inflamed tissues, and up-regulated *CCL3* and *CCL4*, known as CCR5 ligands. Notably, *THBS1*, shown to take part in late NK cell expansion upon TGF-β stimulation (25), was up-regulated along with *TNFRSF1B*, involved in TNFα-mediated NK cell proliferation. Besides, *KLF4*, playing a role in murine NK cell survival and maintenance (26), was also up-regulated. Given the adverse clinical outcome of CD56^neg^ CD16^+^ NK cells expansion in AML, we also explored relevant transcripts related to NK cell cytotoxicity. Among them, *TLR2* and *GZMH* were up-regulated. However, we also found transcripts that could inhibit NK cell-mediated cytotoxicity. CD56^neg^ CD16^+^ NK cells down-regulated *INF2* described as a key factor of T cells immune synapse (27). In addition, *ATF3*, a negative regulator of IFN-γ genes in NK cells (28), was up-regulated along with *CST7*. This molecule is known to be an inhibitor of cathepsins C and H and could promote NK cell split anergy (29) (**Figure 3A**, bottom panel). Enrichment analysis on AML-specific transcripts mostly unveiled that up-regulated transcripts were enriched in cytokine-related transcripts (TNF, IL-12, and type II IFN) whereas down-regulated transcripts were enriched in cell activation-related transcripts (**Figure 3B**).

Then, we sought to identify a specific transcriptomic signature for CD56^neg^ CD16^+^ NK cells based on the overlapping transcripts between AML and HV samples. A set of 57 down-regulated and 108 up-regulated transcripts were found (**Figure 3C**, top panel). We observed the differential expression of cytotoxicity-related transcripts. CD56^neg^ CD16^+^ NK cells up-regulated *LYZ*, the activating receptor *CD86*(30) and the inhibitory receptor *CSF3R* (31). Besides, transcripts from the LILR family (*LILRA1*, *LILRA2* and *LILRB2*) were also up-regulated. On the other hand, CD56^neg^ CD16^+^ NK cells differentially expressed transcripts that could hamper NK cell-mediated killing. Namely, *TMEM163* implied in NK cell degranulation (32) was down-regulated whereas *SIGLEC10*, which is thought to decrease NK cell cytotoxicity in hepatocellular carcinoma (33), was up-regulated. Interestingly, complement transcripts C*3*, *C3AR1*, which was shown to inhibit NK cell cytotoxicity, as well as *C5AR1* with immunoregulatory properties in mice (34) were up-regulated. Next, we investigated transcripts involved in NK cell maturation. CD56^neg^ CD16^+^ NK cells down-regulated transcripts expressed in immature CD56^bright^ NK cells such as *GZMK*(35) or *KIT*(36). They also down-regulated *RUNX2*, repressed during canonical maturation, along with its target genes *ZEB1, BACH2* and *PRDM8*(37). On the other hand, *ZNF683*, known to regulated NK cell development (38) but also involved in NK cell exhaustion in multiple myeloma (39), was up-regulated. Then, we assessed the homing abilities of CD56^neg^ CD16^+^ NK cells. *ITGA1*, a specific marker of NK cell tissue residency (40), was down-regulated along with *CCR7*(41) and *CXCR3*(42). Besides, C*XCR2* and its ligands CXCL8 and *CXCL16*(43), *IL3RA* and *CD4*, increasing cytokine production and cell migration in NK cells (44), were up-regulated (**Figure 3C**, bottom panel). Furthermore, cell activation, cytokine pathways, proliferation and endocytosis were overrepresented among over-expressed transcripts while cell activation, chemotaxis, actin organization and Wnt signaling were overrepresented among under-expressed transcripts as revealed by gene set enrichment analysis from subset-specific transcripts (**Figure 3D**). Together these results strongly suggested that CD56^neg^ CD16^+^ NK cells represent a unique subset of mature NK cells with functional capacities relying on different cytotoxic pathways than conventional NK cells. Notably, some transcripts were related to impaired cytotoxicity or exhaustion, which could partly explain the adverse outcome in AML patients with CD56^neg^ CD16^+^ NK cells.

### Altered phenotype of CD56^neg^ CD16^+^ NK cells in AML patients with CD56^neg^ CD16^+^ NK cells expansion

After RNA-seq profiling, we attempted to confirm our findings with spectral flow cytometry and investigated whether CD56^neg^ CD16^+^ NK cells had a distinct protein expression pattern than conventional NK cells in HV, Non-Expanded and Expanded groups.

**Figure 4:**
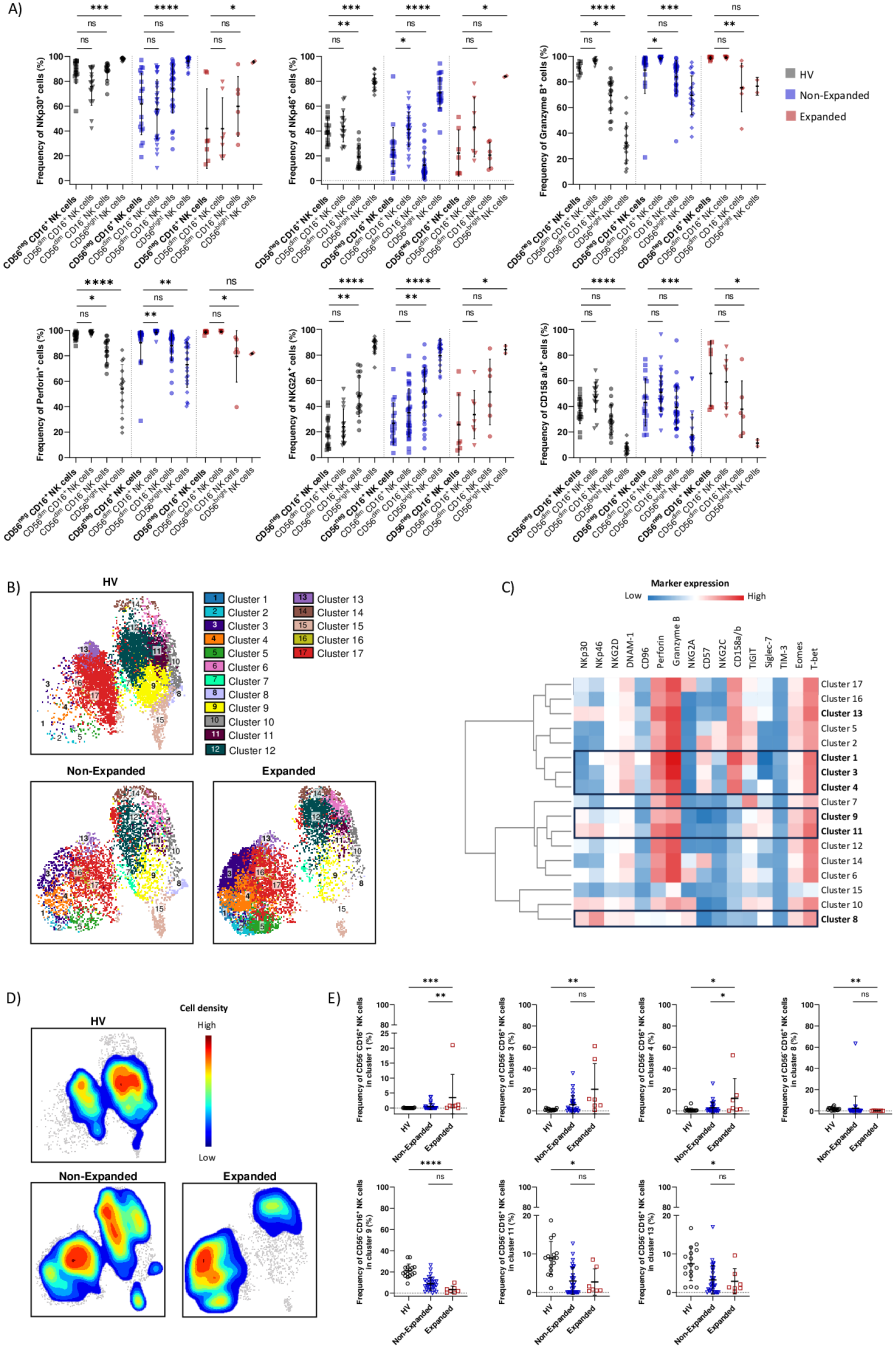
Altered phenotype of CD56^neg^ CD16^+^ NK cells in AML patients with CD56^neg^ CD16^+^ NK cells expansion. **(A)** Boxplots showing frequencies of NK cells markers expression on CD56^neg^ CD16^+^, CD56^dim^ CD16^+^, CD56^dim^ CD16^-^ and CD56^bright^ NK cells in HV, Non-Expanded and Expanded groups. **(B)** UMAP visualization of CD56^neg^ CD16^+^ NK cell clusters defined with FlowSOM in HV, Non-Expanded and Expanded groups. **(C)** Marker expression heatmap of CD56^neg^ CD16^+^ NK cells clusters. Marker expression is normalized, low expression is represented in blue, high expression in red. **(D)** Cell density plot of CD56^neg^ CD16^+^ NK cells in HV, Non-Expanded and Expanded groups. Low cell density is represented in blue, high cell density in red. **(E)** Boxplots showing changes in CD56^neg^ CD16^+^ NK cells repartition among clusters in HV, Non-Expanded and Expanded groups. Statistical significance was determined by Kruskal-Wallis and Dunn *post-hoc* test. P-values below 0.05 were considered significant. *p<0.05, **p<0.01, ***p<0.0001, ****p<0.0001, ns, p not significant. Data is plotted as mean with SD.

CD56^neg^ CD16^+^ NK cells from all three groups had significantly decreased expression of NK cell triggering receptors NKp30 and NKp46 compared to CD56^bright^ NK cells. However, CD56^neg^ CD16^+^ NK cells from the Expanded group expressed similar levels of NKp30 and NKp46 compared to CD56^dim^ NK cells. CD56^neg^ CD16^+^ NK cells from the HV group had decreased NKp46 expression compared to CD56^dim^ CD16^-^ NK cells whereas CD56^neg^ CD16^+^ NK cells from the Non-Expanded group had decreased NKp46 expression compared to CD56^dim^ CD16^+^ NK cells. Regarding direct cytotoxicity, CD56^neg^ CD16^+^ NK cells from the HV and Expanded groups significantly expressed higher levels of granzyme B and perforin compared to CD56^dim^ CD16^-^ NK cells. CD56^neg^ CD16^+^ NK cells from the HV and Non-Expanded groups significantly expressed higher levels of granzyme B and perforin compared to CD56^bright^ NK cells. CD56^neg^ CD16^+^ NK cells from the Non-Expanded group had decreased expression of granzyme B and perforin compared to CD56^dim^ CD16^+^ NK cells. On the other hand, CD56^neg^ CD16^+^ NK cells from the Expanded group had similar expression of granzyme B and perforin than CD56^dim^ CD16^+^ NK cells. Thus, our results are in line with both our transcriptomic and *in vitro* data: CD56^neg^ CD16^+^ NK cells displayed a cytotoxic profile in AML. On the other hand, our results confirmed that CD56^neg^ CD16^+^ NK cells had reached a mature stage as CD56^neg^ CD16^+^ NK cells from all three groups expressed significantly lower levels of NKG2A and higher levels of CD158a/b than CD56^bright^ NK cells. Moreover, CD56^neg^ CD16^+^ NK cells from the HV and Non-Expanded groups had decreased expression of NKG2A compared to CD56^dim^ CD16^-^ NK cells (**Figure 4A**).

Next, we investigated whether CD56^neg^ CD16^+^ NK cells from the Expanded group displayed an altered phenotype compared to the HV and Non-Expanded groups. We performed UMAP analysis of CD56^neg^ CD16^+^ NK cells and automatic clustering using FlowSOM, resulting in 17 clusters, in HV, Non-Expanded and Expanded groups (**Figure 4B**). Clusters were identified by the co-expression of 16 markers (**Figure 4C**; **Supplementary Figure S6**). We plotted CD56^neg^ CD16^+^ NK cells density for each group and observed significant changes in CD56^neg^ CD16^+^ NK cells from the Expanded group compared to the HV and Non-Expanded groups (**Figure 4D**). More CD56^neg^ CD16^+^ NK cells from the Expanded group were found in mature cytotoxic clusters compared to the HV and Non-Expanded groups. Indeed, cluster 1 (NKp46^+^ NKG2D^+^ DNAM-1^+^ CD96^+^ Perforin^+^ Granzyme B^+^ CD57^+^ CD158a/b^+^ TIGIT^+^) and cluster 4 (NKG2D^+^ DNAM-1^+^ Perforin^+^ Granzyme B^+^ CD57^+^ KIR^+^ Siglec-7^+^) were significantly increased in the Expanded group compared to the HV and Non-Expanded groups (**Figure 4E**). Notably, TIGIT was shown to be associated with NK cell exhaustion (45) and TIGIT^+^ NK cells displayed decreased effector function. Besides, CD56^neg^ CD16^+^ NK cells from cluster 3 (NKG2D^+^ DNAM-1^+^ Perforin^+^ Granzyme B^+^ CD57^+^ KIR^+^) were more abundant in the Expanded group than in the HV group (**Figure 4E**). Interestingly, the frequency of terminally mature CD56^neg^ CD16^+^ NK cells expressing NKG2C present in clusters 2 and 5 was not different between the three groups (**Figure 4C**). On the other hand, Siglec-7 expressing clusters, cluster 8 (NKp30^+^ NKp46^+^ NKG2D^+^ DNAM-1^+^ Granzyme B^+^ NKG2A^+^ Siglec-7^+^), cluster 9 (NKp30^+^ DNAM-1^+^ Perforin^+^ Granzyme B^+^ Siglec-7^+^), cluster 11 (NKp30^+^ NKp46^+^ DNAM-1^+^ Perforin^+^ Granzyme B^+^ TIGIT^+^ Siglec-7^+^) and cluster 13 (NKp30^+^ NKp46^+^ NKG2D^+^ DNAM-1^+^ Perforin^+^ Granzyme B^+^ KIR^+^ TIGIT^+^ Siglec-7^+^) were significantly decreased in the Expanded group compared to the HV group. Interestingly, Siglec-7 expression on NK cells were shown to mark fully functional NK cells ligand (46). Therefore, the loss of Siglec-7^+^ CD56^neg^ CD16^+^ NK cells could contribute to adverse clinical outcome of patients with CD56^neg^ CD16^+^ NK cells expansion. Together, our results showed an altered phenotype of CD56^neg^ CD16^+^ NK cells from the Expanded group, with increased mature cytotoxic NK cell expressing inhibitory receptors such as CD158a/b and TIGIT and decreased Siglec-7^+^ NK cells.

## Discussion

In this study, our aim was to further characterize CD56^neg^ CD16^+^ NK cells, a subset that expands in AML. We confirmed that CD56^neg^ CD16^+^ NK cells represent a unique NK cell subset. We used Cytosplore^+HSNE^, an automated clustering approach, on mass cytometry data and observed that CD56^neg^ CD16^+^ NK cells located in NK cells cluster. These results suggested a strong similarity between proteomic phenotype of CD56^neg^ CD16^+^ and conventional NK cells, in line with previous publications (2, 47). Besides, CD56^neg^ CD16^+^ NK cells co-expressed high levels of Eomes and T-bet, distinguishing them from ILCs (48). CD56^neg^ CD16^+^ NK cells seem to be circulating NK cells as they down-regulated transcripts involved in homing or tissue residency such as CCR7 and ITGA1. Interestingly, CD56^neg^ CD16^+^ NK cells from AML patients up-regulated THBS1 and *TNFRSF1B* involved in TGF-β and TNFα-mediated expansion, respectively. Those two cytokines were reported to be increased in AML patients compared to healthy volunteers (49, 50).

We further unveiled that CD56^neg^ CD16^+^ NK cells downregulate *NCAM1*, encoding for CD56, compared to conventional NK cells, suggesting that the loss of CD56 is an intrinsic mechanism. The loss of CD56 was reversible as CD56^neg^ CD16^+^ NK cells could regain CD56 expression when cultivated with IL-2, IL-15 and IL-21. Importantly, CD56^neg^ CD16^+^ NK cells displayed unaltered NK cell function *in vitro* once they regained CD56, known to play a critical role in NK cell cytotoxicity (13).

On the other hand, CD56^neg^CD16^+^ NK cells expansion has been documented in chronic diseases. In a validation cohort, we confirmed that newly-diagnosed AML patients with CD56^neg^ CD16^+^ NK cells expansion had significantly poorer clinical outcomes than others. This affords the possibility for adoptive NK cell therapy or the use of NK-cell engagers on CD56^neg^ CD16^+^ NK cells from AML patients. Importantly, no significant change in CD8^+^ T cells, CD4^+^ T cells, TCRVδ2^+^ T cells and regulatory T cells frequency was found in patients with CD56^neg^ CD16^+^ NK cell expansion.

Bulk RNA-seq and spectral flow cytometry results showed that CD56^neg^ CD16^+^ NK cells poorly expressed NKG2A and up-regulated KIRs. Recently, Rebuffet et al. (51) performed high-dimensional single-cell profiling of human NK cells and identified 3 NK cell subsets in peripheral blood. NK1 et NK3 subsets expressed transcripts of NK cell maturity. We applied NK1 and NK3 signatures to our bulk RNA-seq dataset. Interestingly, CD56^neg^ CD16^+^ NK cells displayed high expression of transcripts from NK3 signature, identifying terminally mature NK cells (**Supplementary Figure S7**). Therefore, our results strongly suggest that CD56^neg^ CD16^+^ NK cell have reached a late maturation stage. Importantly, we observed that CD56^neg^ CD16^+^ NK cells overexpressed *ZNF683*. This transcription factor was recently identified as a driver of NK cell exhaustion in multiple myeloma (39), another hematological malignancy. The authors performed pseudotime analysis on ZNF683^+^ NK cells and underlined 5 transcripts indicative of exhaustion state. Among them, 4 were found up-regulated in CD56^neg^ CD16^+^ NK cells of AML patients, namely *ZNF683*, *LAG3*, *FGL2* and *CST7*, involved in NK cell split anergy (29). Furthermore, AML patients with CD56^neg^ CD16^+^ NK cells expansion displayed increased frequency of terminally mature CD57^+^ KIR^+^ CD56^neg^ CD16^+^ NK cells compared to HV or/and patients without expansion. Interestingly, one cluster contained TIGIT^+^ NK cells, a marker of NK cell exhaustion. We previously showed that CD56^neg^ CD16^+^ NK cells from healthy volunteers expressed slightly higher frequencies of PD-1 compared to CD56^dim^ CD16^+^, CD56^dim^ CD16^-^ and CD56^bright^ NK cells NK cells NK cells (8). However, TIM-3 expression was not increased. Therefore, further investigations are needed to assess CD56^neg^ CD16^+^ NK cells exhaustion. On the other hand, Siglec-7^+^ CD56^neg^ CD16^+^ NK cells were decreased. Siglec-7 was shown to be expressed by terminally differentiated NK cells with high cytotoxic potential. Taken together, our results suggested that AML microenvironment could decrease CD56^neg^ CD16^+^ NK cells cytotoxicity, which may partly explain the negative impact of CD56^neg^ CD16^+^ NK cells expansion in AML on survival.

There were some limitations in the study. RNA-seq analysis was performed on pooled samples and without biological replicates. Therefore, we were likely to find high false positive rates in our differential gene expression analysis. Nevertheless, our results corroborated previous studies (2, 5). Besides, CMV status from AML patients were not available and we cannot exclude the possibility that CD56^neg^ CD16^+^ NK cells expansion is driven by CMV infection (52). However, adaptative CD57^+^ NKG2C^+^ NK cells from AML patients with CD56^neg^ CD16^+^ NK cells expansion were not significantly increased compared to the AML patients without expansion (data not shown).

To conclude, we demonstrated that CD56^neg^ CD16^+^ NK cells represent a distinct NK cell subset that can recover CD56 expression *in vitro* and display NK cell functions, which could be leveraged to counteract the adverse clinical outcome of AML patients with CD56^neg^ CD16^+^ NK cells expansion.

## Data Availability

The original RNA-seq contributions presented in the study are publicly available. This data can be found here: https://www.ebi.ac.uk/biostudies/studies/E-MTAB-14569. Further inquiries can be directed to the corresponding authors.
