# Supplementary material for: CD56neg CD16+ cells represent a distinct mature NK cell subset with altered phenotype and are associated with adverse clinical outcome upon expansion in AML
Source: Front Immunol. 2025 Jan 10;15:1487792. doi: 10.3389/fimmu.2024.1487792 (PMC11760599; doi:10.3389/fimmu.2024.1487792)
Supplement: Supplementary file 1 [file DataSheet1.pdf]

Supplementary information for

$CD56^{neg}$ $CD16^{+}$ cells represent a distinct mature NK cell subset with altered phenotype and are associated with adverse clinical outcome upon expansion in AML.

Julia Wlosik, Florence Orlanducci, Manon Richaud, Clemence Demerle, Amira Ben Amara, Marie-Sarah Rouviere, Philippe Livrati, Laurent Gorvel, Marie-Anne Hospital, Nicolas Dulphy, Raynier Devillier, Norbert Vey, Daniel Olive and Anne-Sophie Chretien

Supplementary Materials and Methods

Supplementary Figures 1 to 7

Supplementary

Material and Methods

Spectral flow cytometry

Cryopreserved PBMCs were thawed in a 37°C water bath, resuspended in RPMI-1640 (Gibco) supplemented with 10% of heat inactivated foetal bovine serum (FBS) (Gibco), centrifugated for 5 min at 1,500 rpm and incubated in a 37°C, 5% CO2 incubator for 30 min. After resting, cells were filtered on a 30 µm pre-separation filter (Miltenyi, Bergisch Gladbach, Germany), centrifugated and incubated in 1X PBS (Gibco) with the viability marker Live/Dead Fixable Blue (Thermo Fisher Scientific) for 15 min at room temperature in the dark. Cells were washed in 1X PBS and centrifugated twice before incubation in Fc block (BD Biosciences), to avoid non-specific binding, and BV421 anti-NKG2A (BD Biosciences, clone 131411) for 15 min at room temperature in the dark. Then a premixed antibody cocktail prepared in Brilliant Stain Buffer Plus (BD Biosciences) to mitigate staining artifacts and containing BB515 anti-NKp46 (clone 9E2/NKp46), BUV395 anti-CD3 (clone UCHT1), BUV737 anti-NKG2D (clone 1D11), BUV805 anti-CD16 (clone 3G8), BV650 anti-NKG2C (clone 134591), BV786 anti-CD158 (clone HP-MA4), and BV786 anti-CD158b (clone CH-L) purchased from BD Biosciences and BV510 anti-CD57 (clone AQ17A04), BV570 anti-CD56 (clone HCD56), BV605 anti-DNAM-1 (clone 11A8), and BV750 anti-TIM-3 (clone F38-2E2) from Biolegend was added and cells were incubated for 25 min at room temperature in the dark. Cells were washed in 1X PBS and centrifugated twice before incubation in PE-Cy5 anti-CD34 (BD Biosciences, clone 581), AF700 anti-SIGLEC-7 (clone 6-434), APC anti-NKp30 (clone P30-15), APC-Fire750 anti-TIGIT (clone A15153G), PE-Cy5 anti-CD14 (clone M5E2), PE-Cy5 anti-CD15 (clone W6D3), PE-Cy5 anti-CD33 (clone WM53), and PerCP anti-CD45 (clone HI30) from Biolegend for 25 min at room temperature in the dark. After washing, cells were fixed and permeabilized with the eBioscience Foxp3 / Transcription Factor Staining Buffer Set (Thermo Fisher Scientific). PerCP-eF710 anti-perforin (clone dG9), and eF450 anti-granzyme B (clone N4TL3) purchased from Thermo Fisher Scientific, and PE anti-EOMES (clone X4-83), and PE-CF594 anti-T-bet (clone O4-46) from BD Biosciences were used for intracellular staining. All used antibodies are listed in Supplementary Table 1. Samples were acquired on a Cytek® Aurora cytometer (Cytek Biosciences, Fremont, CA, USA) equipped with 5 laser and 64 detectors. A control sample was added at every run for batch effect correction. Acquisition, unmixing and data cleaning (debris, doublets and dead cells removal) were performed on SpectroFlo V.3.2.1 (Cytek Biosciences). NK cell subsets were gated following the gating strategy outlined in Supplementary Figure 3. Data analysis was performed on OMIQ platform (http://www.omiq.ai/, Boston, MA, USA).

Cell cloning and functional assays

Due to the scarcity of CD56^{neg} CD16^{+} NK cells, we performed cell cloning using limiting dilution method. CD56^{dim} CD16^{+} and CD56^{neg} CD16^{+} NK cells from one healthy donor and one AML patient were FACS-sorted as previously described and plated in 96-wells round-bottom plate at a concentration of 1, 10, and 100 cells per well in RPMI-1640 (Gibco) supplemented with 10% of heat inactivated FBS (Gibco), 100U/mL IL-2 (Proleukin, Clinigen Healthcare France, Lyon, France), 10ng/mL IL-15 (Miltenyi), and 5ng/mL IL-21 (Biolegend, San Diego, CA, USA). Irradiated K562 cells were used as feeders. Medium was changed and cytokines were added every 2 days. After clonal expansion, clones were phenotyped on day 24 with PE-Vio770 anti-CD56 (clone REA196, Miltenyi), KrO anti-CD3 (clone UCHT1, Beckman Coulter, Brea, CA, USA), FITC anti-CD16 (clone REA423, Miltenyi), PE anti-KIR2D (clone REA1042, Miltenyi), APC anti-NKG2A (clone Z199, Beckman Coulter), and APC-Vio770 anti-CD57 (clone REA769, Miltenyi). 7-AAD (Miltenyi) was used as viability marker. On the same day, NK cell clones were harvested for cytotoxicity and cytokine production assays. NK cells were cocultured with K562 cells at an E:T ratio of 1:1 for 4h at 37°C, washed and incubated with a mix of GolgiStop, FITC anti-CD107a (clone H4A3), and FITC anti-CD107b (clone H4B4) purchased from BD Biosciences. Then, cells were stained for PE-Vio770 anti-CD56 (Miltenyi, clone REA196), V450 anti-CD16 (BD Biosciences, clone 3G8), PE anti-INFγ (Miltenyi, clone 45-15), and APC anti-TNFα (Miltenyi, clone REA656). Cells were fixed and permeabilized with the eBioscience Foxp3 / Transcription Factor Staining Buffer Set (Thermo Fisher Scientific) before intracellular staining. To measure caspase-3/7 activities, K562 target cells were labelled with Cell Proliferation Dye eF670 (Thermo Fisher Scientific) and cocultured with NK cells at E:T ratios of 5:1, 2:1, and 1:1 for 2.5h at 37°C. CellEvent Caspase-3/7 Detection Reagents were added followed by a 30 min incubation at 37°C. All used antibodies are listed in Supplementary Table 1. Data were acquired on a FACSCanto II flow cytometer (BD Biosciences) and analysed on FlowJo V10.8.1.

RNA-sequencing analysis

Bulk RNA-seq data were generated from 8 samples : CD56^{neg} CD16^{+}, CD56^{dim} CD16^{+}, CD56^{dim} CD16^{-} and CD56^{bright} NK cells from HV and AML. FASTQC was applied to the raw sequencing data for quality control. Sequences were aligned to the human reference genome hg38 using STAR. To ensure that the gating strategy of our FACS-sorting did not exclude CD33^{+} NK cells(1), we adapted the bulk RNA-seq NK cell signature from(2) to our dataset and used CIBERSORTx(3) to estimate the abundance of NK cells among the CD13/33/34 cell population based on the LM7 gene signature(4) (Supplementary Figure

5A). Raw count normalization and differential gene expression analysis were performed with limma(5) R package. Normalized average expression >1 and |logFC| > 2 were retained as thresholds. To assess the biological relevance of DEGs, enrichment analysis was performed using Interactive Enrichment Analysis R package.

Mass cytometry

Cryopreserved PBMCs were thawed in a 37°C water bath, resuspended in RPMI-1640 (Gibco) supplemented with 10% of heat inactivated and filtered FBS (Gibco), centrifugated at 20°C for 5 min at 400 x g and incubated in a 37°C, 5% CO2 incubator for 30 min in RPMI-1640 supplemented with 2% heat inactivated FBS and 2.5 U/mL Universal Nuclease 25kU (Thermo Fisher Scientific). Cells were washed and incubated with 1µM cisplatin for viability stain. To avoid nonspecific antibody staining, cells were incubated in Human Fc Block (BD Biosciences) and stained with an extracellular master mix for 45 min at 4°C. Cells were washed and incubated for secondary antibodies for 30 min at 4°C. Before intracellular staining, permeabilization was performed with the Foxp3 Staining Buffer Set (eBioscience, San Diego, CA, USA) for 30 min at 4°C. Cells were washed and fixed with 2% formaldehyde (Thermo Fischer Scientific) and 1:1 000 Cell-ID™ Intercalator-Ir 125 µM (Fluidigm, San Francisco, CA, USA) overnight. After washing, cells were resuspended in deionized water and EQ™ Four Element Calibration Beads. Samples were acquired on a Helios mass cytometer (Fluidigm). All used antibodies are listed in Supplementary Table 1. For more details on clinical samples and staining protocol, please refer to our previous study(6) and our staining protocol(7). After acquisition, raw data were normalized with CyTOF® Software v7.1 (Fluidigm). $CD45^+$ cells were gated following the gating strategy outlined in Supplementary Figure 2. Cells from HV and AML patients were downsampled to 872 796 cells, with Omiq. Dimension reduction and clustering was performed using $Cytosplore^{+HSNE}$ (8).

Statistical Analysis

Prism V.9 was used to conduct statistical analysis. Results were expressed as mean ± SD. Mann-Whitney test was used to compare between two groups and Kruskal-Wallis test followed by Dunn's method for multiple comparisons was used to compare between three or more groups. OS and RFS were calculated using Kaplan–Meier method and log-rank test. P-values < 0.05 were considered significant. All statistical tests are specified in figure legends. *=p<0.05, **=p<0.01, ***=p<0.001, ****=p<0.0001.

References

1. Eckel AM, Cherian S, Miller V, Soma L. CD33 expression on natural killer cells is a potential confounder for residual disease detection in acute myeloid leukemia by flow cytometry. Cytometry B Clin Cytom. 2020 Mar;98(2):174–8.

2. Shembrey C, Foroutan M, Hollande F. A new natural killer cell-specific gene signature predicting recurrence in colorectal cancer patients. Front Immunol [Internet]. 2023 Jan 6 [cited 2024 Nov 7];13. Available from: https://www.frontiersin.org/journals/immunology/articles/10.3389/fimmu.2022.1011247/full

3. Newman AM, Steen CB, Liu CL, Gentles AJ, Chaudhuri AA, Scherer F, et al. Determining cell type abundance and expression from bulk tissues with digital cytometry. Nat Biotechnol. 2019 Jul;37(7):773–82.

4. Tosolini M, Pont F, Poupot M, Vergez F, Nicolau-Travers ML, Vermijlen D, et al. Assessment of tumor-infiltrating TCRVγ9Vδ2 γδ lymphocyte abundance by deconvolution of human cancers microarrays. Oncoimmunology. 2017;6(3):e1284723.

5. Ritchie ME, Phipson B, Wu D, Hu Y, Law CW, Shi W, et al. limma powers differential expression analyses for RNA-sequencing and microarray studies. Nucleic Acids Research. 2015 Apr 20;43(7):e47.

6. Chretien AS, Devillier R, Granjeaud S, Cordier C, Demerle C, Salem N, et al. High-dimensional mass cytometry analysis of NK cell alterations in AML identifies a subgroup with adverse clinical outcome. Proceedings of the National Academy of Sciences. 2021 Jun;118(22):e2020459118.

7. Ben Amara A, Rouviere MS, Fattori S, Wlosik J, Gregori E, Boucherit N, et al. High-throughput mass cytometry staining for deep phenotyping of human natural killer cells. STAR Protoc. 2022 Oct 20;3(4):101768.

8. van Unen V, Höllt T, Pezzotti N, Li N, Reinders MJT, Eisemann E, et al. Visual analysis of mass cytometry data by hierarchical stochastic neighbour embedding reveals rare cell types. Nat Commun. 2017 Nov 23;8(1):1740.

Supplementary Figures

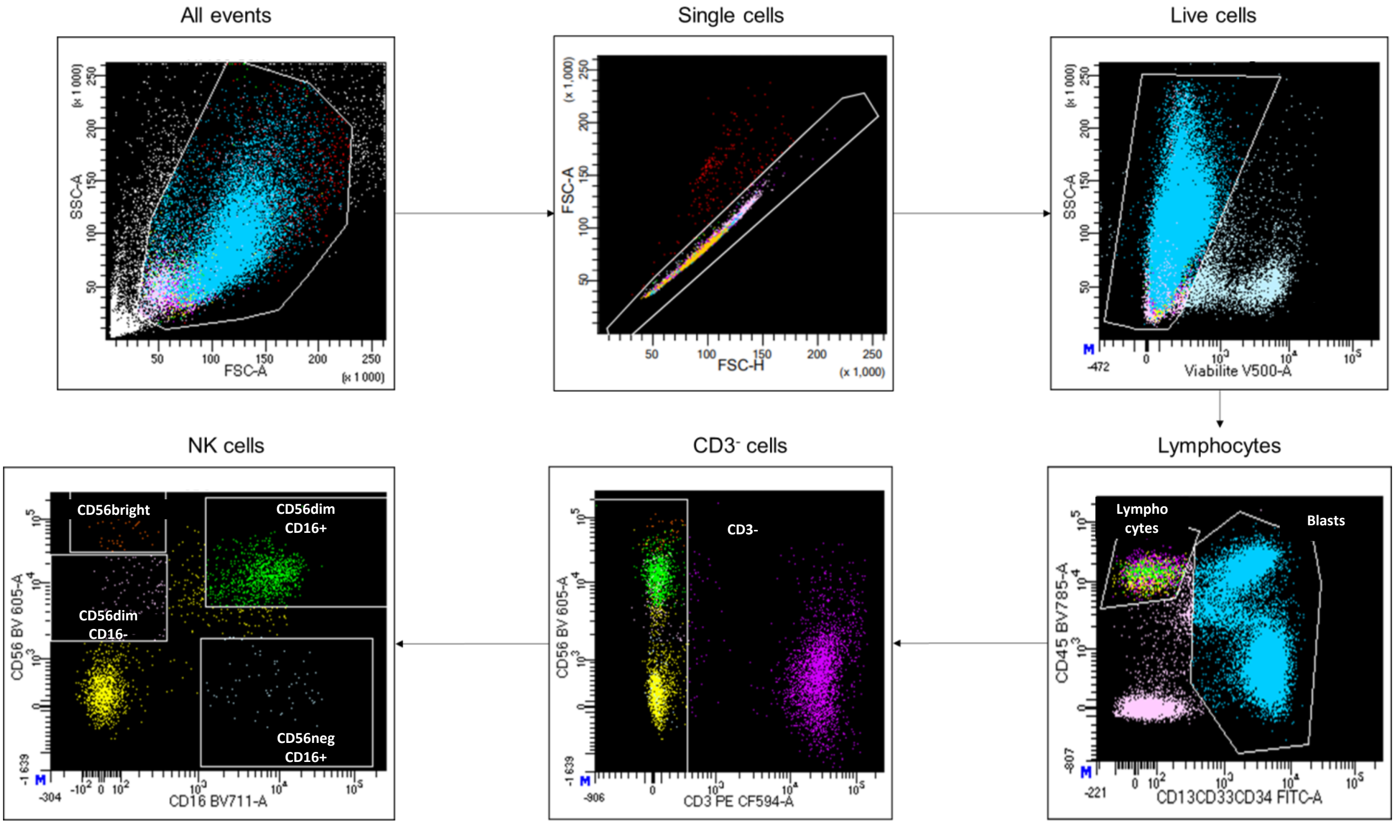

Supplementary Figure 1. Gating for Fluorescence-Activated Cell Sorting (FACS). Gating strategy to discriminate CD56^{neg} CD16^{+}, CD56^{dim} CD16^{+}, CD56^{dim} CD16^{-} and CD56^{bright} NK cells within single, live, CD45^{+} CD13^{-} CD33^{-} CD34^{-} CD3^{-} cells.

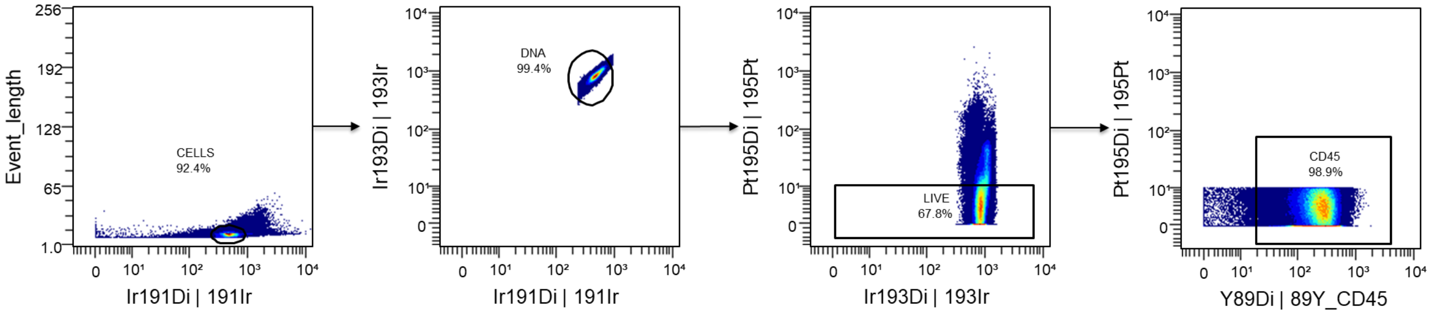

Supplementary Figure 2. Gating for mass cytometry. Gating strategy to discriminate CD45$^+$ cells within cells, DNA, live cells.

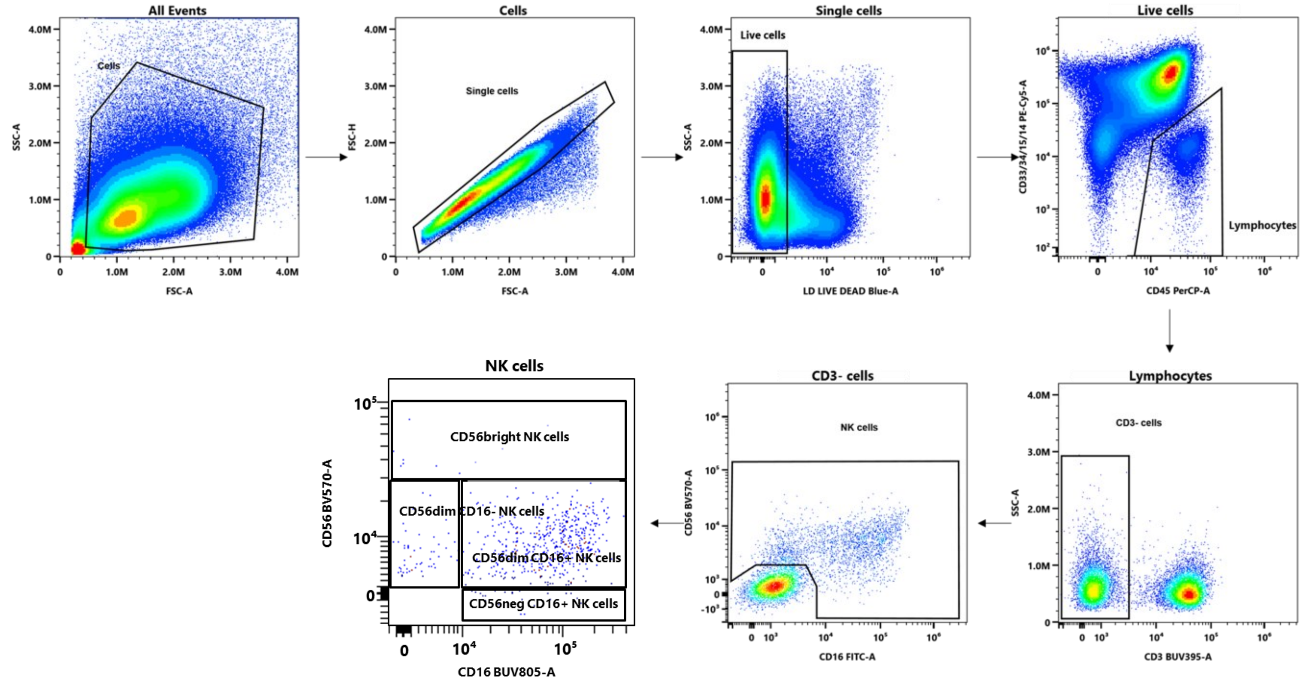

Supplementary Figure 3. Gating for spectral flow cytometry. Gating strategy to discriminate $CD56^{neg}$ $CD16^{+}$, $CD56^{dim}$ $CD16^{+}$, $CD56^{dim}$ $CD16^{-}$ and $CD56^{bright}$ NK cells within single, live, $CD45^{+}$ $CD14^{-}$ $CD15^{-}$ $CD33^{-}$ $CD34^{-}$ $CD3^{-}$ cells.

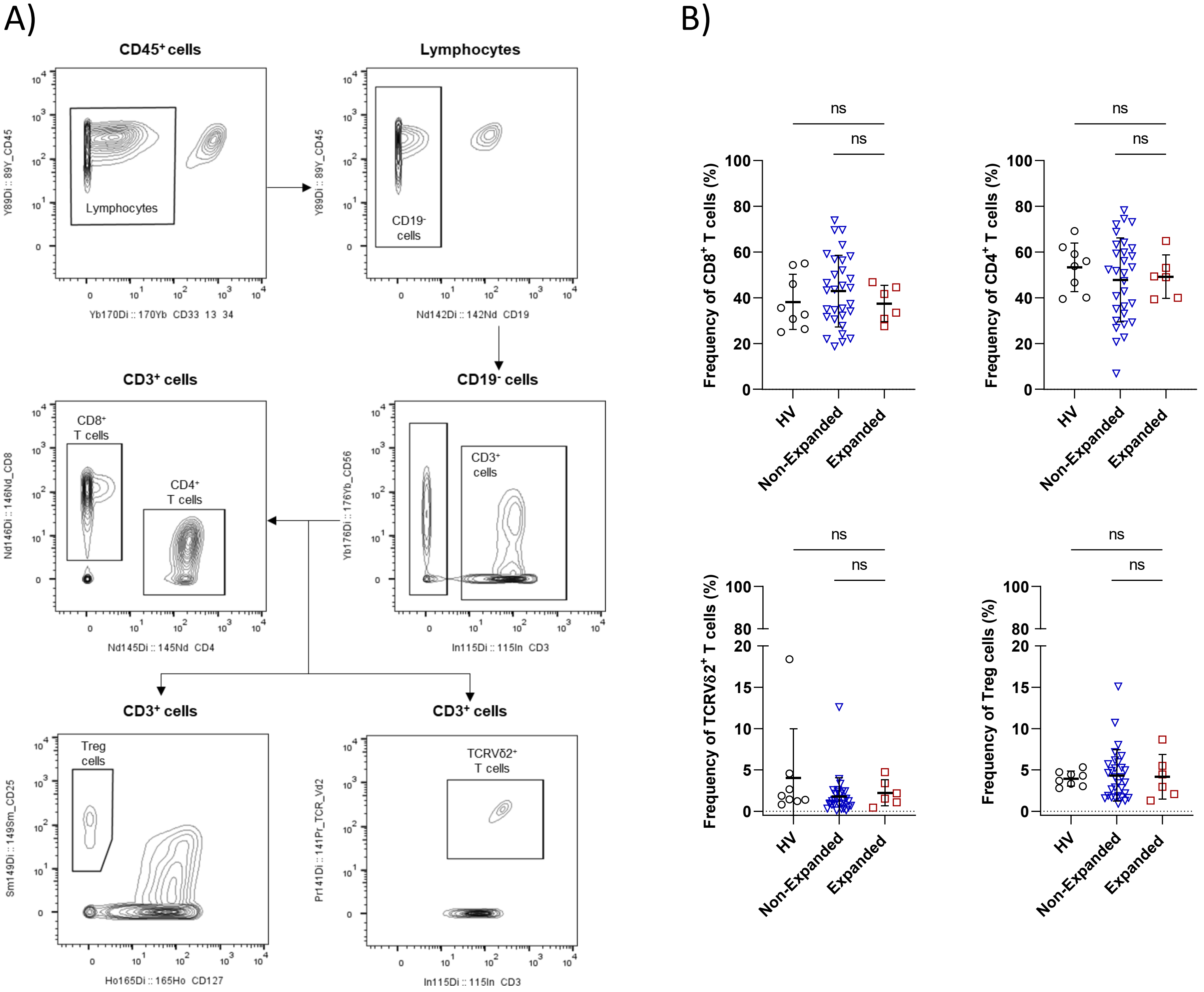

Supplementary Figure 4. Frequencies of T cell populations are not altered in AML patients from the Expanded group compared to HV and AML patients from the Non-Expanded group.

A) Gating strategy for the identification of T cell populations in mass cytometry data. CD8$^+$ T cells were identified as CD45$^+$ CD13$^-$ CD33$^-$ CD34$^-$ CD19$^-$ CD3$^+$ CD8$^+$ cells. CD4$^+$ T cells were identified as CD45$^+$ CD13$^-$ CD33$^-$ CD34$^-$ CD19$^-$ CD3$^+$ CD4$^+$ cells. TCRVδ2$^+$ T cells were identified as CD45$^+$ CD13$^-$ CD33$^-$ CD34$^-$ CD19$^-$ CD3$^+$ TCRVδ2$^+$ cells. Regulatory T cells were identified as CD45$^+$ CD13$^-$ CD33$^-$ CD34$^-$ CD19$^-$ CD3$^+$ CD25^{high} CD127$^-$ cells. B) Boxplots displaying frequencies of T cell populations in the HV, Non-Expanded and Expanded groups. Statistical significance was determined by Kruskal-Wallis and Dunn post-hoc test. P-values below 0.05 were considered significant. *=p<0.05, **=p<0.01, ***=p<0.0001, ****=p<0.0001, ns=p not significant. Data is plotted as mean with SD. Treg: regulatory T cells.

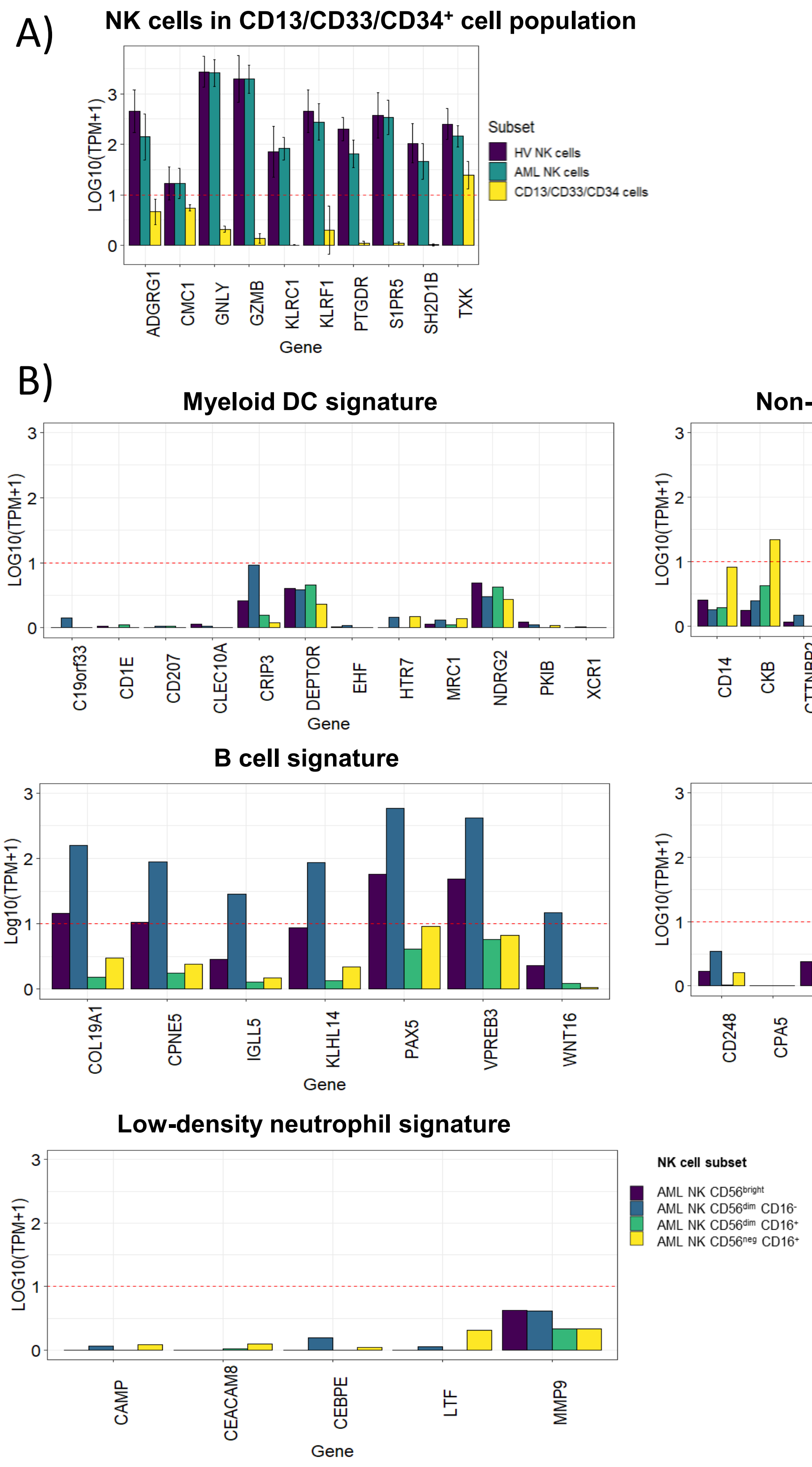

Supplementary Figure 5. Validation of FACS-sorting strategy using RNA-seq data

We insured that $CD33^+$ NK cells were not gated out. A) Normalized gene expression (TPM) of NK cell bulk gene signature adapted from Shembrey et al., *Front. Immunol.* 2022. B) Normalized gene expression (TPM) of cell type-specific transcriptomic signatures for non-classical monocytes, myeloid dendritic cells, B and T cells adapted from the human protein atlas (https://www.proteinatlas.org/) and a low-density neutrophils signature adapted from Montaldo et al., *Nat. Immunol.* 2022 in $CD56^{bright}$ (dark blue), $CD56^{dim}$ $CD16^-$ (blue), $CD56^{dim}$ $CD16^+$ (green) and $CD56^{neg}$ $CD16^+$ (yellow) NK cells.

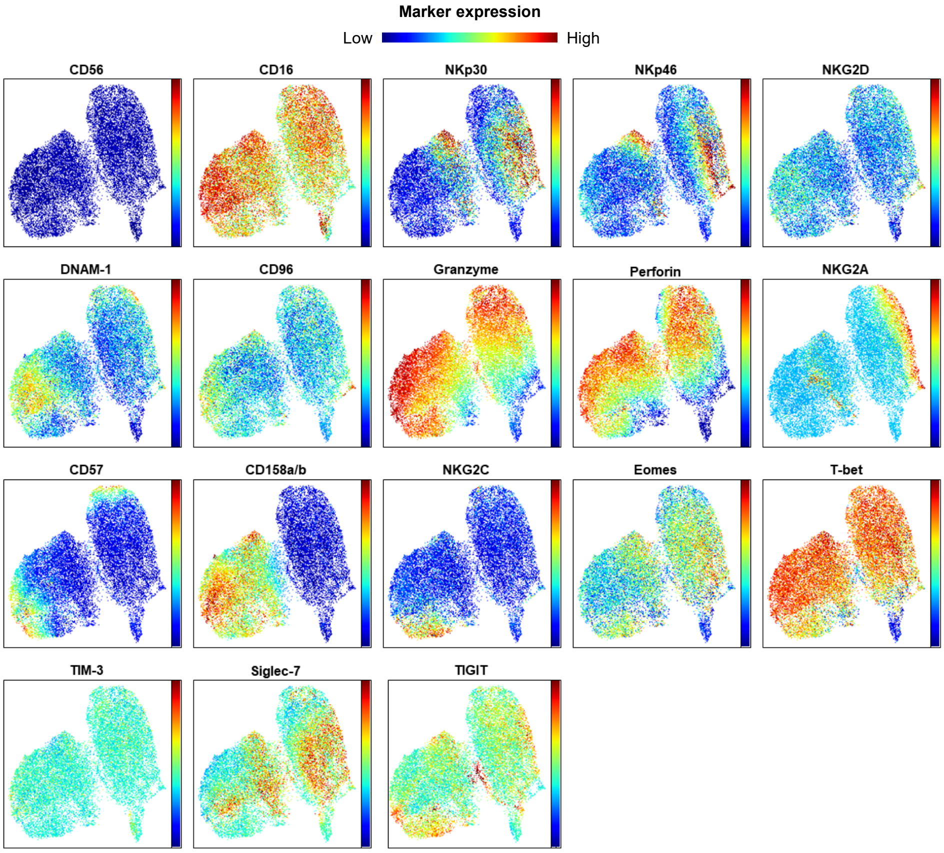

Supplementary Figure 6. UMAP dimension reduction of CD56^{neg} CD16^{+} NK cells from spectral flow cytometry data. PBMCs from N=16 HV and N=38 AML patients at diagnosis were stained with a 27-antibodies panel. The 16 markers represented above were used to defined CD56^{neg} CD16^{+} NK cells clusters. Data showing marker expression intensity were projected on UMAPs. High marker expression is represented in red, low marker expression in blue.

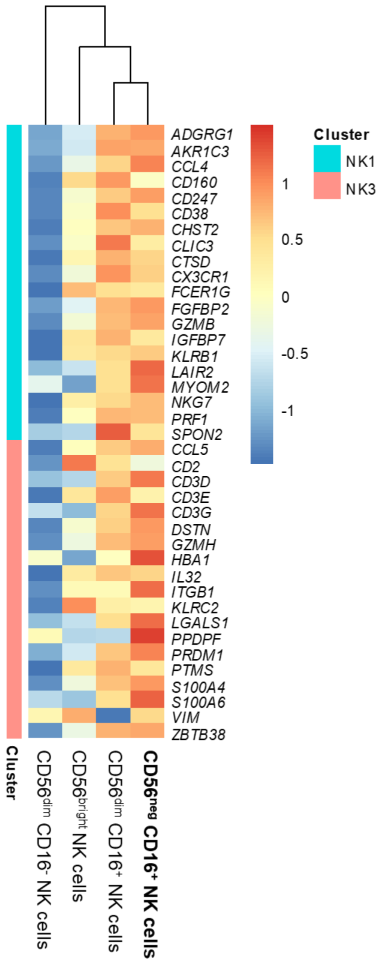

Supplementary Figure 7. Heatmap displaying the top 20 transcripts identifying NK1 and NK3 NK cell subsets from Rebuffet et al., *Nature Immunology* 2024 in NK cell subsets from AML patients. CD56^{neg} CD16^{+} NK cells showed high expression of transcripts defining the NK3 NK cell subset. Expression is shown as z-scores, lowest z-scores are blue and higher z-scores are red.
