# Supplementary material for: CD56neg CD16+ cells represent a distinct mature NK cell subset with altered phenotype and are associated with adverse clinical outcome upon expansion in AML
Source: Front Immunol. 2025 Jan 10;15:1487792. doi: 10.3389/fimmu.2024.1487792 (PMC11760599; doi:10.3389/fimmu.2024.1487792)
Supplement: Supplementary file 2 [file DataSheet2.pdf]

Supplementary Tables

Supplementary Table 1. Lists of antibodies.
Supplementary Table 2. RNA quality control metrics.
Supplementary Table 3. Frequency of $CD56^{neg}$ $CD16^{+}$ NK cells.
Supplementary Table 4. DGEA $CD56^{neg}$ $CD16^{+}$ vs conventional NK cells in AML patients.
Supplementary Table 5. DGEA $CD56^{neg}$ $CD16^{+}$ vs conventional NK cells in HV.

Supplementary Table 1. Lists of antibodies.

FACS				
**Specificity**	**Fluorochrome**	**Clone**	**Reference**	**Vendor**
Viability	Live/Dead Fixable Aqua	-	L34957	Thermo Fisher Scientific
CD3	PE-CF594	UCHT1	562280	BD Biosciences
CD13	FITC	SJ1D1	IM0778U	Beckman Coulter
CD16	BV711	3G8	563127	BD Biosciences
CD33	FITC	HIM3-4	555626	BD Biosciences
CD34	FITC	581	555821	BD Biosciences
CD45	BV785	HI30	563716	BD Biosciences
CD56	BV605	NCAM16.2	562780	BD Biosciences
**Spectral flow cytometry**				
**Specificity**	**Fluorochrome**	**Clone**	**Reference**	**Vendor**
Viability	Live/Dead Fixable Blue	-	L23105	Thermo Fischer Scientific
Fc Receptors	Fc Block	Fc1	564220	BD Biosciences
CD3	BUV395	UCHT1	563546	BD Biosciences
CD14	PE-Cy5	M5E2	301864	Biolegend
CD15	PE-Cy5	W6D3	323014	Biolegend
CD16	BUV805	3G8	748850	BD Biosciences
CD33	PE-Cy5	WM53	303406	Biolegend
CD34	PE-Cy5	581	555823	BD Biosciences
CD45	PerCP	HI30	304026	Biolegend
CD56	BV570	HCD56	318330	Biolegend
CD57	BV510	QA17A04	393314	Biolegend
CD158a	BV786	HP-MA4	752508	BD Biosciences
CD158b	BV786	CH-L	743455	BD Biosciences
DNAM-1	BV605	11A8	338324	Biolegend
EOMES	PE	X4-83	566749	BD Biosciences
Granzyme B	eF450	N4TL3	48-8896-42	Thermo Fisher Scientific
NKG2A	BV421	131411	747924	BD Biosciences
NKG2C	BV650	134591	748165	BD Biosciences
NKG2D	BUV737	1D11	748426	BD Biosciences
NKp30	APC	P30-15	325210	Biolegend
NKp46	BB515	9E2/NKp46	564536	BD Biosciences
Perforin	PerCP-eF710	dG9	46-9994-42	Thermo Fisher Scientific
SIGLEC-7	AF700	6-434	339210	Biolegend
T-bet	PE-CF594	avr-46	562467	BD Biosciences
TIGIT	APC-Fire750	A15153G	372708	Biolegend
TIM-3	BV750	F38-2E2	345056	Biolegend

Clone phenotyping				
**Specificity**	**Fluorochrome**	**Clone**	**Reference**	**Vendor**
Viability	7AAD	-	130-111-568	Miltenyi
CD3	KrO	UCHT1	B00068	Beckman Coulter
CD16	FITC	REA423	130-113-954	Miltenyi
CD56	PE-Vio770	REA196	130-113-313	Miltenyi
CD57	APC-Vio770	REA769	130-111-966	Miltenyi
NKG2A	APC	Z199	A60797	Beckman Coulter
KIR2D	PE	REA1042	130-117-627	Miltenyi
**Degranulation and cytokine production assay**				
**Specificity**	**Fluorochrome**	**Clone**	**Reference**	**Vendor**
Viability	Live/Dead Fixable Aqua	-	L34957	Thermo Fisher Scientific
CD16	V450	3G8	560474	BD Biosciences
CD56	PE-Vio770	REA196	130-113-313	Miltenyi
CD107a	FITC	H4A3	555804	BD Biosciences
CD107b	FITC	H4B4	555800	BD Biosciences
INFγ	PE	45-15	130-113-493	Miltenyi
TNFα	APC	REA656	130-120-063	Miltenyi
**Mass cytometry**				
**Specificity**	**Metal**	**Clone**	**Reference**	**Vendor**
Anti-PE	156Gd	PE001	3156005B	Fluidigm
BCL-2	150Nd	124	15071	Cell Signaling Technology
BCL-Xl	158Gd	-	2762	Cell Signaling Technology
CD13	PE/biotinylated	SJ1D1	A07762	BD Biosciences/Sigma
CD158a/h	168Er	EB6B	A09778	Beckman Coulter
CD158b1/b2j	154Sm	GL183	IM1846	Beckman Coulter
CD16	209Bi	3G8	3209002B	Fluidigm
CD19	142Nd	HIB19	3142001B	Fluidigm
CD27	155Gd	L128	3155001B	Fluidigm
CD3	115In	UCHT1	-	Immunomonitoring department*
CD33	PE/biotinylated	D3HL60	A07775	Beckman Coulter/Sigma
CD34	PE/biotinylated	Immu133	IM1420	Beckman Coulter/Sigma
CD4	145Nd	RPA-T4	3145001B	Fluidigm
CD45	89Y	HI30	3089003B	Fluidigm
CD45RA	143Nd	HI100	3143006B	Fluidigm

Mass cytometry				
**Specificity**	**Metal**	**Clone**	**Reference**	**Vendor**
CD56	176Yb	N901	3176009B	Fluidigm
CD57	172Yb	HCD57	3172009B	Fluidigm
CD8A	146Nd	RPA-T8	3146001B	Fluidigm
CD96	166Er	NK92.39	338402	BioLegend
DNAM-1	164Dy	DX11	130-092-479	Miltenyi
Ki-67	159Tb	Ki-67	350502	BioLegend
NKG2A	165Ho	REA110	130-122-329	Miltenyi
NKG2C	152Sm	REA205	130-122-278	Miltenyi
NKG2D	160Gd	ON72	A08934	Beckman Coulter
NKp30	169Tm	Z25	IM3709	Beckman Coulter
NKp46	162Dy	BAB281	3162021B	Fluidigm
TCRpangd	153Eu	REA591	130-122-291	Miltenyi
TCRVd2	141Pr	IMMU 389	IM1464	Beckman Coulter
* : this antibody was produced by the immunomonitoring department of the IPC.				

Supplementary Table 2. RNA quality control metrics.

Sample	RNA Quantity (ng)	RIN	Yield (Mbases)	% ≥ Q30 bases	Quality Score
AML CD56^{bright} NK cells	6.27	6.9	10,202	96.25	36.37
AML CD56^{dim} CD16^{-} NK cells	13.83	7.1	10,810	96.07	36.34
AML CD56^{dim} CD16^{+} NK cells	24.11	1.4	10,142	96.32	36.385
AML CD56^{neg} CD16^{+} NK cells	13.59	6.1	7,577	96.23	36.37
HV CD56^{bright} NK cells	16.75	9.3	10,114	96.16	36.36
HV CD56^{dim} CD16^{-} NK cells	17.02	8.2	10,223	96.25	36.37
HV CD56^{dim} CD16^{+} NK cells	118.35	9.3	10,938	96.08	36.34
HV CD56^{neg} CD16^{+} NK cells	20.91	8.9	10,559	96.37	36.395

Supplementary Table 3. Frequency of $CD56^{neg}$ $CD16^{+}$ NK cells.

Expanded Group	Non-Expanded Group	HV Group
19.4	3.74	1.96
16.2	3.7	3.71
75.6	8.51	5.23
14.9	0.235	8.55
11.8	0.622	1.83
27.6	0.258	5.28
33.9	3.93	2.56
	4.07	5.06
	1.88	4.63
	1.42	6.77
	2.92	3.32
	0.111	3.95
	4.5	7.43
	0.37	3.49
	0.815	5.12
	0.305	4.86
	0.944	
	1.45	
	0.44	
	5.3	
	0.805	
	1.16	
	6.72	
	0.971	
	1.47	
	0.411	
	1.23	
	6.05	
	3.32	
	2	
	0.837	

Supplementary Table 4. DGEA CD56^{neg} CD16^{+} *vs* conventional NK cells in AML patients.

Gene	logFC	AveExpr	t	P.Value	adj.P.Val	B
SERPINA1	8.69149055	2.70645782	1.98207496	0.09351567	0.88766216	-4.31866254
CSF1R	8.26867831	2.57630991	4.20363224	0.00534339	0.84724185	-1.82429711
FCN1	7.27168378	1.0420962	1.07926555	0.32091116	0.92376156	-5.06185673
TNFAIP2	7.02238654	1.72671836	2.02773557	0.08772107	0.88766216	-4.2904319
LILRA1	6.71199013	1.15282764	2.8985051	0.02658363	0.88766216	-3.18643982
SLC11A1	6.63512559	2.51731431	3.0476262	0.02184997	0.88766216	-3.23169044
LILRB2	6.61670364	3.86427052	3.0957753	0.02052292	0.88766216	-3.32389641
HK3	6.52590997	1.27325134	4.29423296	0.00482619	0.82122341	-1.64154361
C5AR1	6.37232089	1.27493833	2.69076973	0.03510436	0.88766216	-3.4624467
AIF1	6.11717009	1.8798502	2.2922463	0.06063961	0.88766216	-3.97546374
SULF2	5.86178333	1.95110947	1.14496456	0.29475913	0.9146472	-5.25436192
MAFB	5.62020387	2.7310102	3.25390059	0.01674533	0.88766216	-2.99935814
S100B	5.52012014	1.51994979	2.57695954	0.0409696	0.88766216	-3.61180929
PLXNB2	5.48883587	5.10772471	2.63481274	0.03786814	0.88766216	-4.03987341
LRP1	5.44304912	5.23673327	2.22582659	0.06650914	0.88766216	-4.62952353
ADGRE1	5.20680448	2.03215799	13.7054228	7.60E-06	0.08222591	3.55990316
CXCL16	5.09556099	2.629631	2.8079442	0.02998911	0.88766216	-3.56516523
ADAMTSL4	5.07375751	1.3432333	4.24522771	0.00509862	0.82979534	-1.76231962
NEURL1	5.05015291	1.28050749	6.65989292	0.000497	0.56241272	0.43325851
MS4A7	4.91739254	1.50527711	3.74735991	0.00909214	0.88766216	-2.35325829
CDKN1C	4.88184445	2.52109562	12.7740686	1.16E-05	0.08222591	3.7205146
RASGRP4	4.65529496	1.45114324	2.64412465	0.03739264	0.88766216	-3.6737054
CD4	4.40385901	1.49172092	1.19430752	0.27631689	0.91079343	-5.28380789
NCF2	4.35897627	2.37497601	1.9552857	0.09709282	0.88766216	-4.78026831
TBC1D8	4.30357356	2.92328141	2.88650235	0.02701008	0.88766216	-3.57751501
LST1	4.2319038	3.69211708	3.52073363	0.01198046	0.88766216	-2.76946897
TGFBI	4.17481218	3.07380011	1.28358618	0.24546245	0.90081557	-5.60194214
DMXL2	4.16127184	4.66334604	3.30600258	0.01567233	0.88766216	-3.15542619
PLAUR	4.14326238	2.23793386	2.20988962	0.06800268	0.88766216	-4.34671828
LILRA2	4.06653483	2.1036145	2.19449781	0.06947788	0.88766216	-4.35415441
VCAN	4.04163179	1.74615578	0.7919994	0.45777171	0.92873602	-5.75446808
C3	4.03519653	1.24059379	4.15331303	0.00565721	0.84724185	-1.80692218
LRRC25	4.00104713	2.81295549	3.24782208	0.01687565	0.88766216	-3.0387688
CKB	3.88172276	1.21521922	4.16044548	0.0056115	0.84724185	-1.77530336
MYOM2	3.86273213	3.92360282	2.24418116	0.06483079	0.88766216	-4.62346569
EPS8	3.86069136	1.51444535	4.16054303	0.00561088	0.84724185	-1.84202231
PYGL	3.82017004	1.09154353	2.42974113	0.05013366	0.88766216	-3.94351597
CXCL8	3.79876604	2.34377063	2.43139727	0.05001937	0.88766216	-4.24227668
CFD	3.76413513	2.78478785	2.97950034	0.02388876	0.88766216	-3.53384082
FMNL2	3.74407329	1.01049996	2.67514084	0.03585403	0.88766216	-3.62437137
HCK	3.72130337	3.28018154	1.69068019	0.14057258	0.88766216	-5.32915447
CD3G	3.68032106	1.67469081	2.8806859	0.02721937	0.88766216	-3.54746533
PRAM1	3.64509221	3.9737033	3.21229508	0.01765978	0.88766216	-3.25373427
CST3	3.57251765	4.51229242	1.6741798	0.14383869	0.88859053	-5.35003902
PILRA	3.57048945	3.7024858	7.53654225	0.00024972	0.56241272	1.16663441
SLC1A7	3.50612833	2.22089155	2.76001989	0.03197791	0.88766216	-3.82320511

Gene	logFC	AveExpr	t	P.Value	adj.P.Val	B
C19orf38	3.42244107	2.16215036	1.95170669	0.09758099	0.88766216	-4.56627578
TLR2	3.39111407	2.72732213	2.44173786	0.04931194	0.88766216	-4.08512464
LMO2	3.37206357	1.2288327	2.07109842	0.08255365	0.88766216	-4.44394299
KIR3DL2	3.35771619	4.7940693	2.36965443	0.05447016	0.88766216	-4.49177088
SORT1	3.35379627	2.64884668	2.6303158	0.03810006	0.88766216	-3.82255428
TCF7L2	3.34847699	5.35813403	3.21298943	0.01764408	0.88766216	-3.32184015
MN1	3.34111267	2.3694622	5.89743379	0.00096094	0.56241272	-0.13819218
FGD4	3.33092986	1.96825125	1.80852665	0.11924803	0.88766216	-4.97665414
CARD9	3.31873667	1.20586304	3.60555373	0.01079523	0.88766216	-2.49589008
PFKFB4	3.3066196	1.03786124	5.86271671	0.00099172	0.56241272	-0.18018772
MCTP1	3.30608733	2.40951546	2.69363848	0.03496857	0.88766216	-3.79100753
IL3RA	3.28895994	3.00511478	1.3628285	0.22065953	0.90074163	-5.59090715
SPI1	3.25110633	4.61216926	1.44195469	0.19816726	0.90035988	-5.71098941
GPBAR1	3.2266189	1.28483291	5.2757168	0.00172676	0.57039498	-0.68542906
RBM47	3.21120944	1.07908208	1.31068445	0.23671506	0.90074163	-5.30079951
P2RX1	3.19716517	2.45393443	1.56858052	0.16652445	0.89221524	-5.29537513
RIN1	3.18170008	1.82799149	2.81896773	0.02955063	0.88766216	-3.46178222
SLC7A7	3.18021808	2.49412717	1.33347249	0.22957433	0.90074163	-5.5783457
CHST15	3.16851599	2.68587567	1.78131745	0.1238724	0.88766216	-5.16630522
CDH23	3.14596549	3.23768291	2.84098535	0.02869529	0.88766216	-3.6586922
ANPEP	3.13558976	1.8948735	1.10682697	0.30971476	0.92284344	-5.58830775
TRIB1	3.123058	2.05246675	1.5705397	0.16607425	0.89221524	-5.24296428
CD86	3.11825066	1.51520852	1.55908712	0.1687223	0.89221524	-5.1931022
SLC31A2	3.1078766	1.57023507	3.12678194	0.01971476	0.88766216	-2.95997539
TKTL1	3.08939971	3.79618953	2.10612873	0.07860521	0.88766216	-4.85764191
ADGRE2	3.08228643	4.73073201	4.91545712	0.00247961	0.57534534	-1.20034943
CLEC12A	3.07763459	1.91880137	3.4639423	0.01285381	0.88766216	-2.64146471
LAIR2	3.00628967	3.49121723	2.34996811	0.05597458	0.88766216	-4.49234225
ERRFI1	2.9609544	1.90712045	2.96412647	0.02437671	0.88766216	-3.38676332
DAPK1	2.92547794	4.03850153	1.72588713	0.13384116	0.88766216	-5.3322292
ALDH3B1	2.89344686	2.38324218	2.07222598	0.08242348	0.88766216	-4.59613949
RNF144B	2.88473972	2.18606939	2.03088014	0.08733558	0.88766216	-4.74472901
ADAP2	2.88114499	1.61380221	1.46582389	0.19180587	0.89833685	-5.27771804
CCL3	2.86139387	6.23319504	2.00363045	0.09073364	0.88766216	-5.01363179
CSF3R	2.84528049	2.3446517	1.04660381	0.33460742	0.92471072	-5.83325501
LYZ	2.82871784	2.52918337	0.61349934	0.56150551	0.93754162	-5.95814976
PTP4A3	2.81958537	1.60704601	2.11940657	0.07715926	0.88766216	-4.42341812
MNDA	2.79017715	1.11130129	1.41149172	0.20656706	0.90035988	-5.39506593
LRP12	2.78574916	2.00102881	3.24120408	0.01701879	0.88766216	-3.01664546
NFIL3	2.76595953	5.48537455	2.73070908	0.03326337	0.88766216	-3.99275862
SYNGR1	2.7644406	3.71812994	2.80205229	0.03022632	0.88766216	-3.84813317
NLRP3	2.76019593	3.08237566	2.20993288	0.06799858	0.88766216	-4.39966633
CD3D	2.7102781	1.24342103	2.68305345	0.03547238	0.88766216	-3.78557219
NINL	2.68359154	3.36452487	2.19179371	0.06974042	0.88766216	-4.73011008
KIR2DS4	2.67201177	3.78779341	1.6610144	0.14649688	0.88859053	-5.42119507
EMILIN2	2.66037574	3.8079099	1.57500006	0.16505357	0.89221524	-5.50499576
PRSS23	2.64342617	5.42503823	1.54043947	0.17311916	0.89481559	-5.63692833

Gene	logFC	AveExpr	t	P.Value	adj.P.Val	B
SIGLEC10	2.63148799	3.60756686	1.37379472	0.21741	0.90063975	-5.77002231
KIF19	2.62335966	2.08900155	2.38017772	0.05368331	0.88766216	-4.34487167
FGFBP2	2.62187491	6.86492508	1.30127096	0.23972198	0.90074163	-5.91895606
HMOX1	2.60580688	3.6216825	2.77429895	0.031371	0.88766216	-3.85280772
DGKG	2.59298278	1.10833109	1.39772778	0.2104678	0.90035988	-5.3923627
ZNF703	2.57333516	3.35663049	2.23457722	0.06570346	0.88766216	-4.52040663
OSCAR	2.57143984	1.09847628	2.10298978	0.07895105	0.88766216	-4.45203949
CHD5	2.562711	1.22688276	9.13620047	8.32E-05	0.3938483	2.07115766
NECTIN1	2.5514266	4.39605496	1.58529207	0.16272097	0.89137041	-5.55235219
H1F0	2.5503699	1.44378499	2.84444222	0.02856342	0.88766216	-3.52446059
KIR3DL1	2.54468731	4.96970226	1.78305534	0.12357186	0.88766216	-5.31830935
CEP295NL	2.51289319	1.39551799	1.28016341	0.24658738	0.90081557	-5.27229812
IRAK3	2.50482603	4.14505099	1.38869252	0.21306468	0.90035988	-5.65783191
GNAL	2.50271834	2.94251874	1.63975554	0.15088887	0.88866672	-5.31166621
IGF2BP2	2.4991777	2.61302203	3.85549497	0.00799295	0.88766216	-2.27379147
INSR	2.47860109	2.46039955	1.39133484	0.21230224	0.90035988	-5.52273902
DUSP8	2.47831733	6.40290506	2.88167288	0.02718374	0.88766216	-3.78992011
LAYN	2.44856699	1.52137232	2.43622607	0.04968771	0.88766216	-4.10105619
ITSN1	2.44043777	1.99084496	6.06408131	0.00082758	0.56241272	0.00981493
DOCK5	2.42014173	7.16891739	2.12024715	0.07706864	0.88766216	-4.84333494
CEBPB	2.40327228	5.96444193	2.70726274	0.03433129	0.88766216	-4.03350522
ICAM4	2.40316725	1.04031188	1.6318337	0.15255746	0.88866672	-4.62667703
ETS2	2.37316821	4.08669604	3.32125234	0.01537266	0.88766216	-3.09724966
SLC2A6	2.36526376	4.26865219	1.8979259	0.10521861	0.88766216	-5.14240181
ATL1	2.354639	1.16539971	3.25548965	0.01671145	0.88766216	-3.03103678
TIMP1	2.34943299	5.10855185	3.19694544	0.01801078	0.88766216	-3.33145462
STARD8	2.34015565	1.41099943	1.88796917	0.10669654	0.88766216	-4.67303938
CADM1	2.3345725	2.82385508	2.2351399	0.065652	0.88766216	-4.59519302
NCS1	2.33431551	1.04328347	1.8140516	0.11832992	0.88766216	-4.38594896
MROH6	2.32621897	2.16494839	2.6713251	0.03603963	0.88766216	-3.78150874
HES4	2.31940887	3.28970453	2.53498369	0.04338739	0.88766216	-3.89314123
TNFRSF1B	2.31210506	7.83391094	2.86838581	0.02766773	0.88766216	-3.78233364
DDR2	2.27476497	1.07994692	2.65618174	0.03678636	0.88766216	-3.76638386
CSF2RB	2.24083193	3.60259448	0.62839175	0.55232314	0.93754162	-6.39990837
ZNF683	2.23986427	4.00231865	2.79606618	0.03046938	0.88766216	-3.89174894
MT2A	2.23891582	5.44545503	2.17606167	0.07128816	0.88766216	-4.78141219
CXCR2	2.23512908	3.03584396	1.46873766	0.19104234	0.89833685	-5.6581737
RBPMS2	2.22392671	1.14620594	1.60957421	0.15734071	0.88955551	-5.01450527
LRRK2	2.21315626	4.29694674	0.83248626	0.43621881	0.92754161	-6.38552142
METRNL	2.21158736	7.9730039	2.84117044	0.02868821	0.88766216	-3.82719672
MYRF	2.21157277	2.89467878	2.06838384	0.08286791	0.88766216	-4.70480112
CERCAM	2.1855656	3.53171001	1.73349568	0.13242799	0.88766216	-5.32471541
AC025884.1	2.18479449	1.34162886	2.32037982	0.05831746	0.88766216	-4.30178545
ZNF385A	2.16458825	3.545704	1.33498824	0.22910625	0.90074163	-5.60596198
CCL4	2.16342559	7.23777948	1.50118852	0.1827269	0.89671781	-5.6669931
DBN1	2.15342221	2.78727045	1.87875472	0.10808263	0.88766216	-5.01625452
CST7	2.14458503	8.28943178	1.3309084	0.2303681	0.90074163	-5.85443994

Gene	logFC	AveExpr	t	P.Value	adj.P.Val	B
SLC16A6	2.13284191	3.88130752	2.08393116	0.08108435	0.88766216	-4.86225998
F2R	2.12408003	5.24551927	1.34299278	0.22664848	0.90074163	-5.88629148
SORCS2	2.11992268	1.31707547	2.18893003	0.07001956	0.88766216	-4.39140265
GOLM1	2.11824361	3.75415396	1.51253389	0.17989988	0.89671781	-5.60489039
LDLR	2.11762192	6.62002234	2.17091709	0.0718019	0.88766216	-4.79201764
OASL	2.11371863	5.99341723	2.20245306	0.06871136	0.88766216	-4.71975091
AKR1C3	2.11003312	3.8276209	1.37793582	0.21619417	0.90063975	-5.79853796
ATF3	2.10409052	3.73789485	6.28581562	0.00068158	0.56241272	0.19931767
THBS1	2.09501157	5.37540667	2.54537712	0.04277504	0.88766216	-4.26412129
SATB2	2.08439282	1.44751835	2.0118107	0.08969979	0.88766216	-4.7154929
KLF4	2.07702456	6.47074967	1.14687396	0.29402657	0.9146472	-6.09000331
ENC1	2.07502553	6.18238743	2.19766942	0.06917124	0.88766216	-4.74599727
GZMH	2.06231036	7.1387788	1.17977115	0.28164498	0.91137877	-6.05491464
KIR2DL1	2.0618394	3.81798594	1.69100392	0.14050922	0.88766216	-5.39325539
GPR141	2.05385822	2.58734378	1.54149007	0.17286861	0.89481559	-5.46595678
PCDHGB6	2.05186383	2.49050033	1.93468062	0.09993712	0.88766216	-4.93822011
PODN	2.03703234	2.13482188	1.58348504	0.16312825	0.89137041	-5.37781728
CYBB	2.03120629	5.03852592	0.66619031	0.52943879	0.93639033	-6.53742886
PCDHGB5	2.02610243	2.08053018	2.34836273	0.05609915	0.88766216	-4.33863312
C3AR1	2.01621418	3.13455574	1.8194261	0.11744348	0.88766216	-5.14514777
SPTBN4	2.00882332	2.03531849	2.80797131	0.02998802	0.88766216	-3.50599312
PDLIM1	-2.00645807	4.96601713	-1.48774023	0.18613141	0.89671781	-5.50145934
HLA-DOA	-2.00994836	2.54632305	-0.54048942	0.60783673	0.9394976	-6.11831396
E2F5	-2.01916994	3.1333762	-0.97940071	0.36427185	0.92539665	-5.74199191
TCF4	-2.01958508	5.28304541	-0.76347819	0.47340024	0.93303722	-6.26990229
EXD2	-2.01973925	2.07476433	-1.9718437	0.09486598	0.88766216	-4.4107669
MZB1	-2.02690664	1.22853298	-0.4579063	0.66273136	0.94741209	-5.71302036
LGMN	-2.03232219	1.58439584	-0.35998797	0.73087942	0.95231782	-5.54435548
THNSL1	-2.03663659	1.16418286	-1.74878279	0.12963231	0.88766216	-4.46317212
KLRC1	-2.03827589	5.33539809	-1.31143531	0.23647666	0.90074163	-5.71475297
FCRL1	-2.04082432	3.46568709	-0.50800875	0.6291246	0.94299397	-6.35154626
GOLGA2P10	-2.0434244	1.86880214	-1.77259858	0.12539102	0.88766216	-4.56612689
CCR1	-2.04441174	1.74269591	-0.85585916	0.42411573	0.92754161	-5.53165743
NCF1C	-2.04448289	2.28702483	-0.62419017	0.55490434	0.93754162	-5.94731948
TCEAL4	-2.04885459	2.70538316	-1.59623311	0.16027549	0.88955551	-4.91437272
CD19	-2.05232534	2.36084416	-0.43865683	0.67588022	0.94741209	-6.13956721
ID3	-2.0707537	1.19841856	-0.49345768	0.63879061	0.94475302	-5.69633355
USP6NL	-2.07442528	3.97797364	-0.56301922	0.59331104	0.93880752	-6.20532778
VWA7	-2.07482288	1.63842114	-1.38070477	0.21538464	0.90063975	-4.96178499
PRDM8	-2.08164153	4.16297534	-1.12075477	0.30418117	0.92170943	-5.84216182
HAPLN3	-2.08536391	3.3343926	-0.46200606	0.65994753	0.94696238	-5.9031943
LAMA5	-2.08599575	3.06591829	-0.62234921	0.55603765	0.93754162	-6.11497005
AC093890.1	-2.08766835	1.21586439	-0.89339085	0.40520162	0.92754161	-5.14852021
CABLES1	-2.08928707	1.50834812	-0.50716175	0.62968509	0.94299397	-5.54948602
PLEKHG1	-2.09060356	3.31124323	-0.48485911	0.6445392	0.94661347	-6.31046335
BACH2	-2.09174304	5.87576191	-1.47146653	0.19032982	0.89833685	-5.65350183
ZNF135	-2.09306377	2.11190119	-2.4279905	0.05025476	0.88766216	-3.88126027

Gene	logFC	AveExpr	t	P.Value	adj.P.Val	B
PMEPA1	-2.09678107	1.96919246	-0.35334945	0.7356074	0.95333	-5.47136629
RUBCNL	-2.10777421	3.03718215	-0.47828388	0.64895325	0.94661347	-6.30463986
TPCN1	-2.1089838	3.79728916	-1.01423735	0.34864336	0.92471072	-5.85712054
FUT8	-2.10944676	4.68876288	-1.72662491	0.13370349	0.88766216	-5.1381761
CARMIL2	-2.11567903	5.65071383	-2.41535147	0.05113823	0.88766216	-4.31573514
CD79A	-2.11886049	5.87885453	-0.5819542	0.58125927	0.93759696	-6.53794261
TNFRSF10A	-2.12062302	3.11588102	-0.92402126	0.39024079	0.92754161	-5.83705682
PRKN	-2.12908215	1.242548	-1.1413572	0.29614731	0.91623767	-5.06440337
UST	-2.13224998	4.25191615	-2.38946982	0.05299837	0.88766216	-4.18300553
UBASH3A	-2.14139313	1.25679486	-1.99386606	0.0919834	0.88766216	-4.39453056
NSFP1	-2.14239013	1.23019105	-1.35140398	0.22409132	0.90074163	-4.77660674
TNRC18P1	-2.14833755	1.12322461	-0.40486464	0.69926449	0.94823758	-5.725349
HLA-DMB	-2.15272831	3.72907977	-0.62573883	0.55395207	0.93754162	-6.16657276
ABCB4	-2.15572986	1.73413372	-0.59161526	0.57516606	0.93754162	-5.95107772
EFHC2	-2.15616144	3.20942541	-1.09896649	0.31287452	0.92376156	-5.50695954
SLC9A7	-2.15959414	3.63184998	-0.46574028	0.65741705	0.94669851	-6.17043466
AC004057.1	-2.16005009	3.25467004	-3.17808706	0.01845244	0.88766216	-2.87504345
TPD52	-2.16052901	2.3548795	-1.39099642	0.21239976	0.90035988	-5.04922815
FHL1	-2.16216805	2.72078723	-0.83716969	0.43377369	0.92754161	-5.67083067
CCR5	-2.16564735	1.25135124	-1.50146347	0.1826579	0.89671781	-4.93009765
TNFRSF13C	-2.17082402	3.49244818	-0.48342065	0.64550352	0.94661347	-6.20015297
DLL1	-2.17996261	4.37287976	-1.26715626	0.25090377	0.90248532	-5.52914801
EPHA4	-2.18522777	5.76744156	-1.691775	0.14035841	0.88766216	-5.27201252
HDAC9	-2.18593792	4.39237141	-1.12443415	0.30273318	0.92156208	-5.80095878
ST14	-2.19287566	2.32395995	-0.35867336	0.73181468	0.95231782	-5.91892623
RASGRP3	-2.20466504	2.67286788	-0.68282272	0.5195635	0.93493607	-6.05876845
ADAM19	-2.21367916	4.14803527	-0.58044483	0.58221465	0.93759696	-6.33698478
RAB3IP	-2.21919856	2.03425996	-1.79299531	0.12186655	0.88766216	-4.5856181
RASGRF2	-2.21955269	3.52343202	-2.27486466	0.06212196	0.88766216	-4.26054114
COL19A1	-2.22114977	2.611114	-0.49378662	0.63857123	0.94475302	-6.20098729
AFF3	-2.2213285	5.54233976	-0.51539266	0.62424988	0.94185446	-6.49407907
SEMA7A	-2.22577665	4.4447137	-0.65386617	0.53683315	0.93754162	-6.14689749
HLA-DQA2	-2.23760717	3.5543656	-0.86469871	0.41960325	0.92754161	-6.0892548
GRAP	-2.24030632	2.62176799	-1.71638991	0.13562564	0.88766216	-4.83853047
FCER2	-2.24126463	1.62311246	-0.48315228	0.64568351	0.94661347	-6.03683257
CRIP2	-2.24686941	1.78713713	-0.55465937	0.59867754	0.9394976	-5.46025306
SLC17A9	-2.24847115	3.61994415	-1.95053491	0.09774135	0.88766216	-4.64515784
PCDH9	-2.25201612	2.47779828	-0.49231146	0.63955535	0.94475302	-5.91169538
ABCG1	-2.25235916	4.79458367	-1.19229907	0.27704789	0.91079343	-5.69900533
NCF1B	-2.25576055	2.25412027	-0.60685234	0.56563379	0.93754162	-5.8597746
SEC22B4P	-2.28334992	1.31148594	-1.59982308	0.1594807	0.88955551	-4.62484223
CCSER1	-2.28346028	1.4322111	-0.40128557	0.701763	0.94823758	-5.7883558
FMN1	-2.28852705	2.1579657	-1.23820671	0.26074872	0.9043141	-5.23355439
AMPD3	-2.29588075	4.12377535	-0.92017237	0.39209727	0.92754161	-5.96282001
ITGA3	-2.3017403	1.40857345	-2.57276204	0.04120484	0.88766216	-3.61570852
SIGLEC17P	-2.31829146	2.91240362	-1.27448631	0.24846322	0.90095422	-5.30325506

Gene	logFC	AveExpr	t	P.Value	adj.P.Val	B
FCRL2	-2.32435724	1.66254981	-0.49659015	0.63670313	0.9443416	-6.00797642
GPR183	-2.33413497	5.03773808	-0.64400809	0.54279474	0.93754162	-6.17490721
ADGRA3	-2.34618168	2.99338438	-2.14350399	0.07460395	0.88766216	-4.19313101
TRABD2A	-2.35139261	1.3882132	-0.8783286	0.41271514	0.92754161	-5.48664778
NEIL1	-2.35989134	4.69339535	-2.4007202	0.05218126	0.88766216	-4.16741257
CCDC141	-2.3621242	4.42189898	-1.12045816	0.30429815	0.92170943	-5.86506518
ADAM28	-2.36675836	5.26701168	-1.00079446	0.3546098	0.92510649	-6.04287133
COBLL1	-2.37156538	3.502272	-0.46975888	0.65469939	0.94669851	-6.22999984
SLC18B1	-2.38419841	2.19379231	-1.23192396	0.2629292	0.90513257	-5.29751627
TMEM163	-2.38447924	1.10975509	-1.43509142	0.20003197	0.90035988	-4.72042308
IGSF9B	-2.3904198	2.37317466	-1.71469024	0.13594742	0.88766216	-4.75163925
PTPRS	-2.39127708	3.17248252	-0.29837781	0.775226	0.95904178	-5.80443199
NAV2	-2.39528918	1.34165793	-1.30819841	0.2375059	0.90074163	-5.00041669
PAX5	-2.40874262	4.94136891	-0.5800984	0.58243406	0.93759696	-6.43770847
TTN	-2.4133425	8.54793053	-1.39417532	0.21148539	0.90035988	-5.81818249
CNKSR2	-2.43511158	1.52494552	-0.50679082	0.62993063	0.94299397	-5.64202567
MICAL3	-2.43639244	6.32049898	-1.61259047	0.1566843	0.88926755	-5.4700131
TAS2R43	-2.44023319	1.50822331	-2.08954053	0.08045046	0.88766216	-4.01926932
POU2AF1	-2.44600971	3.53359887	-0.5895658	0.57645547	0.93754162	-6.29161741
PCSK6	-2.44844152	1.5815584	-1.37854269	0.21601651	0.90063975	-4.95803509
AL121768.1	-2.45283301	2.74397054	-1.88124294	0.10770658	0.88766216	-4.60229742
PEG10	-2.45804613	1.17431967	-1.41603108	0.2052951	0.90035988	-5.03188139
BTLA	-2.4760692	1.0210026	-0.53888313	0.60887997	0.9394976	-5.81247405
CD22	-2.48035702	3.8772744	-0.59463602	0.57326869	0.93754162	-6.33179289
RALGPS2	-2.50028088	4.92381036	-0.84593652	0.42922354	0.92754161	-6.14987479
CPNE5	-2.50367233	1.38532274	-0.57583211	0.58514008	0.93767504	-5.80840333
CASK	-2.50465898	4.87727195	-1.83520815	0.11487805	0.88766216	-5.02519737
ATP1B1	-2.50629927	2.36986835	-0.68246157	0.51977666	0.93493607	-5.31644316
STRBP	-2.51897584	4.26986879	-1.03128115	0.34119436	0.92471072	-5.87515621
PTPRK	-2.5201048	1.56388981	-0.46016514	0.66119682	0.94738614	-5.87926902
EBF1	-2.52168424	3.12027258	-0.48857218	0.6420535	0.94515118	-6.14826193
SELL	-2.52489216	7.01665736	-2.23229339	0.06591276	0.88766216	-4.67470186
LARGE2	-2.52895176	1.28820368	-0.5662674	0.5912334	0.93880752	-5.73084896
CCND1	-2.52922225	1.03178621	-1.06873529	0.32527591	0.92389989	-5.0907053
MYO1E	-2.53758456	4.25337559	-1.26384536	0.25201301	0.90277581	-5.56876911
BANK1	-2.53957211	4.49813113	-0.60216711	0.56855467	0.93754162	-6.37477116
FAM129C	-2.54756604	4.01680658	-0.54439376	0.60530519	0.9394976	-6.32176969
LRP5	-2.55660204	1.62820474	-0.46933193	0.65498785	0.94669851	-5.60380867
TTC24	-2.57280583	1.64048379	-1.56262319	0.16790048	0.89221524	-4.76185576
TLR10	-2.57905183	1.24382766	-0.57763822	0.58399361	0.93767504	-5.72479438
MS4A1	-2.64316243	3.6734875	-0.6022359	0.56851172	0.93754162	-6.2729193
OSBPL10	-2.65096896	2.6900588	-0.5338294	0.61216873	0.9394976	-6.09445491
BLK	-2.67676038	3.50658816	-0.59448499	0.57336347	0.93754162	-6.21316239
KIT	-2.6770686	2.54434807	-0.64003978	0.54520624	0.93754162	-5.81610654
SYNPO	-2.67975541	1.34412593	-0.50516298	0.6310088	0.94299397	-5.6976359
INF2	-2.68753497	3.95130316	-0.82756779	0.43879739	0.92754161	-5.8934249
INPP4B	-2.69239496	2.03528694	-0.5374047	0.60984104	0.9394976	-5.81582643

Gene	logFC	AveExpr	t	P.Value	adj.P.Val	B
ARHGAP42	-2.70671851	2.98634653	-4.0377504	0.00645881	0.85410725	-1.90441481
LTB	-2.70713364	5.47035029	-1.07645181	0.32207271	0.92376156	-6.03678207
PTK2	-2.71372271	4.22173607	-0.82807818	0.4385293	0.92754161	-5.86202079
RCAN3	-2.71494577	1.78968119	-0.47468716	0.65137438	0.94661347	-5.58730476
CCR7	-2.71960423	3.35824642	-0.56968177	0.58905402	0.93850963	-6.04803795
BLNK	-2.72352438	1.75094496	-0.51380639	0.62529537	0.94185446	-5.93844248
FAM111B	-2.73898167	1.53458894	-0.97269872	0.36734094	0.926471	-5.47661726
CELSR1	-2.80268042	4.5975704	-0.94611367	0.37971474	0.92754161	-5.94159942
CDHR1	-2.80585929	3.29527404	-0.67986415	0.52131134	0.93493607	-5.692337
COL1A1	-2.81343507	1.2086694	-0.49141849	0.64015146	0.94475302	-5.06765906
FCRL5	-2.82724399	2.07276265	-0.46805829	0.65584875	0.94669851	-5.82639229
RUNX2	-2.82986253	4.67136446	-0.92020978	0.39207919	0.92754161	-5.85501166
MYC	-2.83228388	4.89241052	-1.07798879	0.3214378	0.92376156	-5.90712737
MYO7A	-2.87560366	1.21988689	-0.42480731	0.6854185	0.94741209	-5.43142247
SH2D3A	-2.88565199	3.91225467	-2.28652367	0.06112356	0.88766216	-4.19194592
ZEB1	-2.89164888	5.60122606	-1.74470951	0.13037157	0.88766216	-5.13120895
JHY	-2.90551825	2.31773634	-1.59984267	0.15947637	0.88955551	-4.66134903
SLC4A10	-2.91582006	2.11697202	-1.27984286	0.24669296	0.90081557	-4.94866407
AXIN2	-2.92084208	1.21200841	-0.52252889	0.61955827	0.93974126	-5.5335289
PATJ	-2.93120065	5.56088465	-2.32873649	0.05764562	0.88766216	-4.39405777
KLF8	-2.96492184	3.14592984	-2.04735892	0.08534327	0.88766216	-4.37436803
PPP1R9A	-2.98333	4.65433259	-1.48695289	0.18633254	0.89671781	-5.3551781
HVCN1	-3.01337099	3.86856269	-1.1061519	0.30998508	0.92306813	-5.7349373
ANK3	-3.01914333	4.37497975	-2.14025403	0.07494348	0.88766216	-4.53204021
HOOK1	-3.02313672	1.98970958	-1.37981918	0.21564325	0.90063975	-4.79431432
KIF26B	-3.03965682	1.06855094	-0.33737728	0.74703431	0.95349998	-5.19733831
COL4A3	-3.04381413	1.48888028	-0.49131545	0.64022026	0.94475302	-5.56622147
GSTM1	-3.04392002	1.09291513	-1.33053761	0.23048308	0.90074163	-4.71233618
SCML1	-3.04758903	2.63464065	-0.4530676	0.66602451	0.94741209	-5.30726171
STYK1	-3.12235205	1.09632921	-0.74732753	0.48241248	0.93475642	-5.31540092
LEF1	-3.12285333	3.80299814	-1.33871198	0.22795994	0.90074163	-5.40370382
STAP1	-3.1389498	3.42859533	-1.14261252	0.29566362	0.91608981	-5.46276864
NCAM1	-3.14910389	7.16076196	-2.10694074	0.07851599	0.88766216	-4.79889853
CAPG	-3.16628685	3.16658008	-0.61258707	0.56207101	0.93754162	-5.87215421
BEND5	-3.19601146	1.36373083	-0.70624175	0.50586316	0.93493607	-5.20333989
NELL2	-3.21578667	2.86618094	-0.70430852	0.50698501	0.93493607	-5.86229279
CXCR3	-3.27155137	5.13392062	-1.55156217	0.1704838	0.89398678	-5.13510235
DPP4	-3.31776422	1.27231053	-0.42252627	0.68699561	0.94741209	-5.29458733
NBEA	-3.39158429	1.93184211	-0.54229112	0.60666779	0.9394976	-5.47570655
CARMIL1	-3.41070961	2.3208972	-0.72605363	0.49446149	0.93493607	-5.53041993
CRACR2B	-3.44607676	1.01157396	-1.0651692	0.32676501	0.92389989	-4.88534761
IL7R	-3.45454067	5.51816664	-1.11089647	0.30808933	0.92257091	-5.94436469
LSR	-3.50801222	3.38761047	-0.859085	0.42246486	0.92754161	-5.70991997
GZMK	-3.51428388	4.2401577	-0.90701119	0.39849633	0.92754161	-5.78678589
MCF2L	-3.61206778	1.67568376	-1.84034312	0.1140553	0.88766216	-4.26850545
BTBD6P1	-3.63293569	2.02577173	-1.01208446	0.34959347	0.92471072	-5.32489865
CD27	-3.74087289	1.67862489	-0.83004527	0.43749716	0.92754161	-5.38703029

Gene	logFC	AveExpr	t	P.Value	adj.P.Val	B
CNR2	-3.92214982	3.203277	-1.13558199	0.29838118	0.91649243	-5.34484324
PAWR	-3.96092027	1.91791235	-0.62812294	0.55248806	0.93754162	-5.31743725
DTX1	-3.9735793	3.2813977	-0.68129635	0.52046477	0.93493607	-5.38064253
ITGA1	-3.98791723	1.73954671	-1.46880272	0.19102533	0.89833685	-4.65336263
ZMAT4	-4.00441376	2.05762259	-0.60045199	0.56962618	0.93754162	-5.06742349
COL4A4	-4.02471324	1.87902254	-0.69328668	0.51341231	0.93493607	-5.42402934
CEP170B	-4.12849419	1.53317077	-0.67789009	0.52247968	0.93493607	-5.12343167
IGFBP4	-4.20412592	1.84908834	-0.40668342	0.69799639	0.94823758	-5.27616025
TNFRSF11A	-4.46758888	3.63856035	-0.67678885	0.52313217	0.93493607	-5.43625123
ANO9	-4.72127308	3.24982344	-0.81807073	0.4438075	0.92754161	-5.33884738
PACSIN1	-7.41149371	1.8934766	-0.32989217	0.75241377	0.95407326	-4.88713297

Supplementary Table 5. DGEA CD56^{neg} CD16^{+} *vs* conventional NK cells in HV.

Gene	logFC	AveExpr	t	P.Value	adj.P.Val	B
C5AR1	7.25087623	1.27493833	4.38730145	0.00435249	0.42969876	-1.57157132
CSF1R	6.98986827	2.57630991	3.82135988	0.00832315	0.47075135	-2.27372321
SERPINA1	6.31531912	2.70645782	3.10502225	0.02027821	0.65775843	-3.32876492
MPO	6.28958076	1.93251582	4.61368103	0.00340328	0.40271576	-1.48073335
MS4A7	6.20939766	1.50527711	4.05660886	0.00631976	0.45108492	-1.90161926
SLC11A1	6.12832874	2.51731431	3.43823372	0.01327197	0.56841965	-2.81269537
CDKN1C	6.12777952	2.52109562	16.4368647	2.55E-06	0.01810077	4.58690664
MAFB	6.12475292	2.7310102	4.26052128	0.00501179	0.4350768	-1.83205724
CFD	6.0345903	2.78478785	3.95232626	0.00713278	0.46271901	-2.11095697
LILRA1	6.004158	1.15282764	4.02046908	0.00658922	0.45108492	-1.99425949
NEURL1	5.97200268	1.28050749	8.46622241	0.00012896	0.20704456	1.53438663
LILRB2	5.95915472	3.86427052	3.06185448	0.0214484	0.67085675	-3.38777383
HK3	5.90010076	1.27325134	4.50936876	0.00380827	0.40325118	-1.42962094
CKB	5.6295681	1.21521922	8.43229547	0.00013196	0.20704456	1.64015094
HMOX1	5.58496726	3.6216825	5.5531512	0.00132173	0.33599908	-0.475021
ADAMTSL4	5.45459803	1.3432333	3.85192648	0.00802678	0.47075135	-2.12128539
AIF1	5.45393433	1.8798502	3.81613162	0.00837505	0.47075135	-2.36622477
SIGLEC10	5.44969743	3.60756686	2.54528536	0.0427804	0.84414054	-4.03685977
CXCL16	5.31457445	2.629631	3.74682719	0.00909795	0.48229333	-2.46801591
C3	5.28897462	1.24059379	6.32039849	0.00066157	0.26854148	0.20668411
CXCL8	5.28845556	2.34377063	2.84704276	0.02846465	0.73056451	-3.48168168
SPI1	5.15619164	4.61216926	2.22834652	0.06627609	0.94241961	-4.57215036
ZNF703	5.12535792	3.35663049	4.73184504	0.00300189	0.40271576	-1.32960018
LMO2	5.05383974	1.2288327	3.36450106	0.01455634	0.59255585	-2.75151168
ADGRE1	5.04717935	2.03215799	17.337915	1.85E-06	0.01810077	4.84592568
LRRC25	4.98536544	2.81295549	4.85323681	0.00264415	0.3871301	-1.19563384
PLAUR	4.93832792	2.23793386	3.38037656	0.01426871	0.58588294	-2.90876164
CHST15	4.92717569	2.68587567	2.45204828	0.04861699	0.87152523	-4.08256739
RASGRP4	4.84050177	1.45114324	3.32250725	0.01534828	0.60156838	-2.86485444
SLC7A7	4.83784184	2.49412717	2.32657659	0.05781849	0.92503075	-4.334717
LRP1	4.80449988	5.23673327	2.03735155	0.08654763	0.99649919	-4.86012751
CD86	4.78263676	1.51520852	2.47641209	0.04701529	0.86297958	-3.94765619
TNFAIP2	4.78191425	1.72671836	2.63574079	0.03782046	0.8149915	-3.90646048
RNF144B	4.75869544	2.18606939	3.05571871	0.02162058	0.67085675	-3.22090253
LILRA2	4.69154279	2.1036145	3.25880039	0.01664108	0.60967915	-3.05745729
NCF2	4.68855406	2.37497601	2.39191303	0.05281979	0.89140486	-4.2317695
TBC1D8	4.57869275	2.92328141	3.39071651	0.01408471	0.58338635	-2.92743943
FCN1	4.56082648	1.0420962	2.01594254	0.08918211	0.99649919	-4.78155105
PLXNB2	4.48490701	5.10772471	2.29472371	0.06043133	0.92525081	-4.50920633
EMILIN2	4.46765942	3.8079099	2.58984814	0.0402561	0.82408995	-4.03720589
ANPEP	4.45335352	1.8948735	2.34038376	0.0567226	0.91783362	-4.31625488
FMNL2	4.44798335	1.01049996	3.26112621	0.01659185	0.60967915	-2.84336368
LST1	4.44203491	3.69211708	4.23964255	0.00513074	0.4350768	-1.92924692
PYGL	4.41649356	1.09154353	3.07077884	0.0212006	0.67085675	-3.11246026
TRIB1	4.30607205	2.05246675	2.45079607	0.04870084	0.87152523	-4.10867047
DOK3	4.2902032	2.70428594	1.9351514	0.09987121	0.99649919	-4.80565851

Gene	logFC	AveExpr	t	P.Value	adj.P.Val	B
PDK4	4.28232147	1.81248633	3.94002164	0.00723616	0.46285855	-2.07157257
P2RX1	4.26241509	2.45393443	2.40031756	0.05221027	0.8893901	-4.23591777
HCK	4.228597	3.28018154	1.82820399	0.11600972	0.99649919	-5.03664388
CD3G	4.21846761	1.67469081	2.68167412	0.0355386	0.79992414	-3.61302481
SNED1	4.1526379	2.94696281	1.7814729	0.12384549	0.99649919	-5.07725727
CYBB	4.14171123	5.03852592	1.28687422	0.24438606	0.99649919	-5.82350021
TCF7L2	4.11268254	5.35813403	3.86018767	0.00794871	0.47075135	-2.40857292
PFKFB4	4.06114441	1.03786124	7.9107168	0.0001899	0.20753133	1.23770023
H1F0	4.03205896	1.44378499	3.91471314	0.00745406	0.46651891	-2.07056252
TMEM63C	4.01438502	1.06528441	4.01570567	0.00662568	0.45108492	-1.94542621
RBM47	3.93465254	1.07908208	2.0800761	0.08152293	0.98795597	-4.49167235
STARD8	3.91719957	1.41099943	3.89719676	0.00760914	0.46904596	-2.16257746
SLC2A6	3.84758113	4.26865219	2.84577376	0.0285128	0.73056451	-3.69141146
DAPK1	3.71915719	4.03850153	2.15925326	0.07298073	0.96719888	-4.66268124
LYL1	3.70809481	2.51778698	1.81878698	0.11754855	0.99649919	-4.89552941
ALOX5	3.69594653	4.62862221	1.24236436	0.25931443	0.99649919	-5.85740608
YES1P1	3.69478151	1.15026084	1.76695852	0.12638304	0.99649919	-4.74623959
MGLL	3.68853989	1.12415959	3.29309724	0.01593092	0.60554288	-2.93977276
CD4	3.63417737	1.49172092	1.89867204	0.10510868	0.99649919	-4.93378949
SLC29A1	3.60383423	2.17655299	5.55366335	0.00132109	0.33599908	-0.44346665
IFNGR2	3.58986772	4.15360586	1.22741255	0.26450465	0.99649919	-5.88494999
RRAS	3.5886702	1.97009487	7.6048876	0.00023734	0.24084873	1.14512329
ARHGEF11	3.52374234	2.18884134	3.03530401	0.02220433	0.68163681	-3.31441214
CARD9	3.5204199	1.20586304	3.60302073	0.01082869	0.53385199	-2.4357151
MMP17	3.5128035	1.37231864	1.56937896	0.16634084	0.99649919	-5.09158648
DMXL2	3.50721216	4.66334604	2.88462583	0.02707741	0.73056451	-3.69679922
SULF2	3.5059973	1.95110947	1.60075644	0.15927468	0.99649919	-5.4160023
CDH23	3.49081096	3.23768291	3.59330728	0.01095808	0.53385199	-2.70709826
IL3RA	3.48247836	3.00511478	1.73097584	0.1328944	0.99649919	-5.23155154
MARCKS	3.43571236	3.28229916	1.42925122	0.20163135	0.99649919	-5.57831836
TGFBI	3.4357058	3.07380011	1.43975901	0.19876207	0.99649919	-5.64114479
JUP	3.43166375	3.40000664	1.73099509	0.13289083	0.99649919	-5.16406237
TIAM2	3.43158221	2.88121597	3.52417565	0.01192967	0.5501591	-2.74659413
FGD4	3.42409482	1.96825125	1.90480332	0.10420966	0.99649919	-4.80813767
LRRK1	3.4217293	3.45194031	1.21670865	0.26827535	0.99649919	-5.79362751
OSCAR	3.42001421	1.09847628	2.55462639	0.04223776	0.83926124	-3.73004498
GRK3	3.41603017	4.02767147	1.38446615	0.21428935	0.99649919	-5.67095208
CAMKK1	3.37643864	2.17064758	2.4500552	0.04875052	0.87152523	-4.14628401
ADAP2	3.3550145	1.61380221	2.01227484	0.08964148	0.99649919	-4.65437324
PTP4A3	3.34277359	1.60704601	3.00536546	0.02309146	0.68163681	-3.3105028
CAMK1	3.33863215	1.88379183	4.2752331	0.00492983	0.4350768	-1.77165931
TIMP2	3.33069013	1.58185936	2.31406622	0.05883044	0.92525081	-4.29085081
ETS2	3.32876041	4.08669604	4.98918546	0.00229939	0.3871301	-1.1005482
CD3D	3.28831582	1.24342103	2.25462944	0.06389495	0.93523463	-4.04381736
SLC15A3	3.28471379	4.24988157	1.77955617	0.12417772	0.99649919	-5.21488475
PRAM1	3.27949399	3.9737033	3.0164279	0.02275927	0.68163681	-3.49567286
TIMP1	3.25667756	5.10855185	4.48094721	0.00392781	0.40436551	-1.67870062

Gene	logFC	AveExpr	t	P.Value	adj.P.Val	B
PECAM1	3.2552339	6.65608891	3.98735731	0.00684736	0.45217319	-2.26435537
MN1	3.24830265	2.3694622	6.34268922	0.00064903	0.26854148	0.24262541
PDLIM7	3.24738175	3.32225965	3.06758732	0.02128886	0.67085675	-3.38574974
NR1D1	3.24588652	4.71144569	6.04191886	0.00084404	0.29605402	-0.06560156
RIN1	3.23703828	1.82799149	3.89494062	0.00762937	0.46904596	-2.2426286
LRRK2	3.23316305	4.29694674	1.14172712	0.29600471	0.99649919	-5.95601403
KYNU	3.22979429	2.62859205	1.45421225	0.19487664	0.99649919	-5.44196606
INSR	3.21201723	2.46039955	2.02545514	0.0880017	0.99649919	-4.74291972
CEP295NL	3.19500806	1.39551799	2.67395523	0.03591159	0.80345825	-3.81449957
BTK	3.19238248	3.96931014	1.179311	0.28181507	0.99649919	-5.86580513
CSF3R	3.1838812	2.3446517	1.45965917	0.19343057	0.99649919	-5.50971035
DGKG	3.17628749	1.10833109	1.45068841	0.19581745	0.99649919	-5.12289349
NDST1	3.15592674	2.34223565	2.23477149	0.06568569	0.94194527	-4.41934411
AC025884.1	3.14948392	1.34162886	2.22613485	0.06648059	0.94241961	-4.06919581
S100B	3.10176985	1.51994979	2.63804188	0.03770253	0.8149915	-3.89748894
FGD2	3.07927351	4.76720685	1.14943389	0.29304684	0.99649919	-5.97297477
HSPA6	3.0768889	2.93775338	8.63339483	0.00011527	0.20704456	1.56947403
EPS8	3.07598083	1.51444535	3.63476512	0.01041746	0.52696878	-2.47212307
ALDH3B1	3.07371668	2.38324218	2.66468928	0.03636485	0.8063169	-3.8819074
CPVL	3.07271783	1.37584238	1.50984042	0.18056732	0.99649919	-5.49184684
FCGR3A	3.07149671	6.23881449	1.5377804	0.17375482	0.99649919	-5.55568496
TYMP	3.05661005	4.06113091	2.98238071	0.02379852	0.69046588	-3.52195146
RAB31	3.05194766	2.4041434	1.87776179	0.10823305	0.99649919	-4.9486461
SLC31A2	3.04510106	1.57023507	5.15156197	0.00195234	0.37482199	-0.8582807
PPARGC1B	3.00302136	3.20699008	3.44636617	0.0131381	0.56561522	-2.85609024
CST3	2.99220315	4.51229242	1.55615935	0.16940561	0.99649919	-5.53390165
MYO7B	2.94458312	1.0222808	0.84019154	0.43220134	0.99649919	-5.60197139
HAL	2.93062773	1.77328224	3.0379572	0.0221275	0.68163681	-3.1845964
ANXA5	2.92071334	4.94921564	3.3417717	0.01497928	0.59778259	-3.07806416
MNDA	2.91251426	1.11130129	1.20488754	0.27249343	0.99649919	-5.38701379
EVI5	2.90302725	3.45393382	2.04078817	0.08613212	0.99649919	-4.77452879
ITSN1	2.89175307	1.99084496	6.95316105	0.00039174	0.25990797	0.70678787
KCNQ1	2.88438258	2.42691684	1.07360371	0.32325196	0.99649919	-5.90433201
COTL1	2.8537629	5.79806393	1.32582905	0.23194775	0.99649919	-5.81758666
C19orf38	2.84109802	2.16215036	2.63355018	0.0379331	0.8149915	-3.97723636
MT2A	2.82877278	5.44545503	2.69190129	0.03505073	0.79802206	-3.97675
MYOM2	2.8231364	3.92360282	1.58993375	0.16167921	0.99649919	-5.41556124
ABCA1	2.82027777	4.70918035	1.08984928	0.31657282	0.99649919	-6.0829935
MCTP1	2.81193477	2.40951546	2.55051785	0.04247553	0.84280711	-4.01744304
FAM110A	2.79827812	4.78816318	8.95515956	9.34E-05	0.20704456	2.1174416
CLEC12A	2.78108812	1.91880137	4.2690085	0.00496433	0.4350768	-1.80876902
TAGLN	2.77970894	2.35654315	2.34799815	0.05612749	0.91772345	-4.24063832
PPM1N	2.77356042	1.27408876	4.21364888	0.00528327	0.4350768	-1.75541753
PNKD	2.73632929	3.22578962	9.42947717	6.92E-05	0.20704456	2.35688595
NPL	2.72571656	1.32337323	3.38525868	0.01418151	0.58399028	-2.68361815
NLRP3	2.70437091	3.08237566	3.01041872	0.02293907	0.68163681	-3.51314231
CEBPB	2.70097647	5.96444193	3.00717511	0.02303676	0.68163681	-3.54783352

Gene	logFC	AveExpr	t	P.Value	adj.P.Val	B
NRXN2	2.69741897	1.78886457	2.2173869	0.06729582	0.94660567	-4.33260721
PILRA	2.68405953	3.7024858	6.71225087	0.00047604	0.26011985	0.51722989
GNB4	2.66723695	2.1098586	1.3649729	0.22002068	0.99649919	-5.55137273
CD300C	2.64964212	1.57738587	2.78018802	0.03112428	0.74819407	-3.57958581
JPT1	2.6294202	4.4206235	10.8465764	3.05E-05	0.14454849	3.12312494
S100A11	2.61839769	5.069629	3.60293838	0.01082978	0.53385199	-2.74794991
C3AR1	2.61499007	3.13455574	2.19325316	0.06959859	0.95439655	-4.50651211
ARAP3	2.60985155	3.98770368	2.96176877	0.02445249	0.69898704	-3.5322597
MPEG1	2.60552773	4.61782718	0.81098765	0.4475708	0.99649919	-6.31654714
MROH6	2.59577052	2.16494839	3.28482498	0.01609913	0.60554288	-2.9825302
SOD2	2.5882934	5.66544869	4.68009589	0.00317072	0.40271576	-1.4614448
ITPRIPL2	2.58121299	1.3443912	3.20327921	0.01786503	0.62993316	-2.9105982
NAPSB	2.55376222	2.54783372	0.79555176	0.45585088	0.99649919	-6.12940367
CEBPA	2.54661151	4.08779001	2.58711334	0.04040639	0.82597639	-4.07062774
DBP	2.54276749	3.67089727	5.20551882	0.00185044	0.37482199	-0.78800306
SLC30A1	2.54087223	4.20014829	4.70477196	0.00308892	0.40271576	-1.40569649
KRT72	2.51693917	2.1497234	1.49556544	0.18414332	0.99649919	-5.29817663
ZNF385A	2.51240052	3.545704	2.02866528	0.08760692	0.99649919	-4.89215224
LILRB1	2.51030501	6.08946217	1.74082377	0.13108061	0.99649919	-5.2943378
REPS2	2.50886145	1.6423051	1.49781587	0.18357523	0.99649919	-5.24229663
PLEKHO2	2.49497633	4.80042324	3.46850465	0.01278113	0.55795114	-2.91690334
SLC1A7	2.48132916	2.22089155	1.43961908	0.19880004	0.99649919	-5.26825586
GABBR1	2.44893606	4.39913936	1.34860724	0.22493869	0.99649919	-5.74351898
ZNF467	2.44239925	1.72755596	1.32847477	0.23112374	0.99649919	-5.56867427
SORT1	2.44217022	2.64884668	2.51842796	0.04438197	0.85830679	-4.14293697
CHD5	2.42060079	1.22688276	6.72166543	0.00047238	0.26011985	0.38211707
CX3CR1	2.41926657	5.30994158	1.21439619	0.26909605	0.99649919	-5.86880115
JAML	2.40042024	1.87266038	1.45094281	0.19574939	0.99649919	-5.30624538
CDC42BPB	2.39668477	3.67343806	1.05444862	0.33127514	0.99649919	-6.06052818
GAB2	2.3938174	5.10152614	1.80526792	0.11979283	0.99649919	-5.21624706
TKT	2.38582852	7.24666393	3.75103002	0.0090522	0.48229333	-2.55360674
WARS	2.37920872	6.21715053	6.83582227	0.00043046	0.25990797	0.61188282
CPPED1	2.3746799	3.2406507	2.71733561	0.03386809	0.7834558	-3.80887533
TESC	2.37223445	3.20065304	8.01519208	0.00017626	0.20753133	1.49351184
CBFA2T3	2.36822753	3.81561383	0.78557319	0.46126099	0.99649919	-6.24193661
FCGRT	2.36335891	3.26239782	1.50488135	0.18180222	0.99649919	-5.54382483
TNFSF13B	2.34884463	1.64452748	2.67990176	0.03562388	0.79992414	-3.62721931
SCO2	2.34778301	3.055951	3.01832049	0.02270296	0.68163681	-3.43586602
SAMD4A	2.34702661	1.31972357	1.03623981	0.33905137	0.99649919	-5.56225906
VCAN	2.34595843	1.74615578	0.80457639	0.4509968	0.99649919	-6.20585597
IQSEC2	2.34510055	3.46322347	3.2964962	0.01586237	0.60554288	-3.10852514
ATP6V0A1	2.34117928	2.3300232	0.91666868	0.3937931	0.99649919	-5.91885101
CERCAM	2.33554718	3.53171001	1.63492335	0.1519046	0.99649919	-5.27234141
PTGS1	2.33373992	2.53548755	1.84461324	0.11337555	0.99649919	-4.96007032
CORO1B	2.32232145	3.65832032	2.99936971	0.02327369	0.68163681	-3.48699288
MAP3K20	2.31674847	2.28334276	2.70490967	0.03444047	0.78777532	-3.69402692
ACSS2	2.31008443	1.56989826	2.01710399	0.08903713	0.99649919	-4.49780008

Gene	logFC	AveExpr	t	P.Value	adj.P.Val	B
FGD6	2.30520618	2.85369047	2.94903675	0.02486612	0.70465742	-3.51975907
KCNH3	2.3026266	1.37460429	3.78075202	0.0087358	0.47431526	-2.25242675
IFITM3	2.30075388	5.40453596	1.77413881	0.12512143	0.99649919	-5.25947429
LAG3	2.29560204	3.09877327	1.47309367	0.18990613	0.99649919	-5.30089246
CMKLR1	2.29024937	3.45339293	1.28665934	0.24445628	0.99649919	-5.61982126
TLR7	2.2784043	2.35999236	0.74475981	0.48385606	0.99649919	-6.12756276
PRSS23	2.26376218	5.42503823	1.27748596	0.24747051	0.99649919	-5.85825089
CXCR2	2.26083636	3.03584396	1.1464717	0.29418078	0.99649919	-5.72063699
TMEM273	2.25299811	1.85805805	1.28368465	0.24543015	0.99649919	-5.22784281
KCNN4	2.25058162	1.05124902	0.74781814	0.48213699	0.99649919	-5.74292111
PTPN6	2.24463263	7.76671391	3.61495765	0.01067199	0.53385199	-2.73930881
ANXA2P2	2.23868877	1.31693022	2.63004527	0.03811406	0.8149915	-3.62878082
MTSS1	2.23809159	7.67707384	2.06816858	0.08289288	0.99046185	-4.84712103
MBOAT1	2.23241383	2.30239762	1.28205228	0.24596602	0.99649919	-5.59876036
TNS3	2.23092337	2.9761752	0.72501866	0.49505281	0.99649919	-6.20072446
ACRBP	2.22278129	1.12695784	2.49649071	0.04573696	0.85830679	-3.79634636
PSAP	2.20866885	9.21591723	2.70384959	0.03448978	0.78777532	-3.90773282
CR1	2.20843155	3.3635872	0.6808192	0.52074671	0.99649919	-6.22829271
SEMA4A	2.20348998	3.1558167	1.26418129	0.25190027	0.99649919	-5.8133582
RNF207	2.18912816	1.51094808	1.49263417	0.18488572	0.99649919	-5.25865132
KRT73	2.17828495	3.36287944	1.63765703	0.15132918	0.99649919	-5.25080897
IMPDH1	2.1757214	5.54733758	4.21930408	0.00524966	0.4350768	-1.99478753
PDGFRB	2.17551663	5.45387973	1.23848722	0.26065174	0.99649919	-5.91441119
VSIR	2.17352884	7.32376079	4.2283208	0.00519656	0.4350768	-1.9694923
DGKK	2.16635664	3.72417905	1.11869019	0.3049962	0.99649919	-5.89787922
LYZ	2.16509954	2.52918337	0.85285622	0.4256568	0.99649919	-6.27987191
RYR1	2.15265721	1.95475229	4.27416893	0.00493571	0.4350768	-1.68557745
PCDH1	2.1495433	4.35445948	1.15747092	0.28998882	0.99649919	-5.93331009
PLCL1	2.14119832	1.81255221	2.8090477	0.0299449	0.73858894	-3.53722725
HPGD	2.14057857	1.68984209	1.82207276	0.11700936	0.99649919	-4.76993017
TBXAS1	2.14033005	5.17709634	2.37998988	0.05369725	0.90281283	-4.4133996
RAB3D	2.13987967	2.52535077	2.8389962	0.02877146	0.73056451	-3.59639768
FADS2	2.12719421	3.79741714	1.93916574	0.09931099	0.99649919	-4.96467448
FGFBP2	2.12540449	6.86492508	1.06055395	0.32870047	0.99649919	-6.12071461
RTL5	2.12535303	3.22812078	5.37205929	0.00157198	0.3544938	-0.67547946
GLUL	2.12436649	6.34398577	5.32101763	0.00165193	0.36670264	-0.77954492
FAM214B	2.12378174	4.60154171	5.15803083	0.00193979	0.37482199	-0.9355744
CALHM2	2.1234134	3.90918219	2.56557544	0.04161093	0.83576844	-4.03849385
HSPA7	2.12291364	3.6584197	2.05986445	0.08386205	0.9936849	-4.70815273
PLK3	2.11321063	5.27302038	2.21033245	0.06796072	0.94903868	-4.66595793
BASP1	2.10725089	1.45079371	0.65681211	0.53505968	0.99649919	-5.95667852
RRAGD	2.10307532	1.36961737	1.72187507	0.13459221	0.99649919	-4.94898925
FCGR2C	2.0996054	4.61151258	2.47409428	0.04716525	0.86351789	-4.27026423
CLCF1	2.08538236	2.58879984	2.8721896	0.02752824	0.73056451	-3.56558462
PALLD	2.08027138	4.2638051	1.29512362	0.24170381	0.99649919	-5.77686212
E2F1	2.08018566	2.53423454	2.65990354	0.03660132	0.8063169	-3.81967019
BCL2A1	2.07444107	4.14074815	6.0003819	0.0008759	0.29605402	-0.10431727

Gene	logFC	AveExpr	t	P.Value	adj.P.Val	B
ANXA2	2.07393683	6.16979617	2.35455291	0.05562039	0.91352477	-4.46413155
ZFYVE21	2.07051182	1.14513761	4.10634181	0.00596882	0.45108492	-1.82930407
CTNNA1	2.06972478	5.52349051	1.49150555	0.18517231	0.99649919	-5.61904669
AKR1C3	2.05868459	3.8276209	1.19410851	0.27638925	0.99649919	-5.83040535
GPR25	2.05287694	1.29424759	1.46564552	0.1918527	0.99649919	-4.88448105
NAMPTP1	2.04590615	1.94031736	5.5469352	0.00132953	0.33599908	-0.48088948
MAPK7	2.04534834	5.21708028	4.45559516	0.004038	0.41271892	-1.70601873
KIF19	2.03111058	2.08900155	1.25113291	0.25631199	0.99649919	-5.40560503
FCRL6	2.02760869	5.39011762	1.29076687	0.24311713	0.99649919	-5.83740521
RHOC	2.02352491	4.05987923	2.26137777	0.06329793	0.93423187	-4.54609644
ZNF683	2.02128018	4.00231865	2.18511193	0.07039353	0.95793186	-4.55586648
BMF	2.02102876	2.59933019	1.23955674	0.26028224	0.99649919	-5.72297571
MAPKAPK3	2.01842918	5.91738642	3.68816353	0.00976405	0.50260103	-2.6557898
OSGIN1	2.01672518	1.11672255	3.99399931	0.00679469	0.45108492	-1.95330206
CD180	2.0142426	2.18165411	0.49891755	0.6351545	0.99649919	-5.97805835
MDGA1	2.0104944	3.33559908	1.87539217	0.10859287	0.99649919	-4.72558096
AC004551.1	2.00964029	3.85551851	3.59953774	0.01087489	0.53385199	-2.66173422
CLMN	2.00038479	2.11427633	0.71484668	0.50088973	0.99649919	-6.11232112
GOLGA2P10	-2.00853554	1.86880214	-1.81138197	0.11877268	0.99649919	-4.49726023
SYNGR3	-2.00902378	1.90779628	-2.56609394	0.04158149	0.83576844	-3.57252496
ID1	-2.01373785	1.85815588	-1.32162978	0.23326099	0.99649919	-5.5677522
MED12L	-2.02182943	2.51512967	-1.09208119	0.31566415	0.99649919	-5.66710158
SYNPO	-2.02561488	1.34412593	-0.28829999	0.78257424	0.99649919	-5.47989142
PRDM8	-2.02797247	4.16297534	-1.00563304	0.35245298	0.99649919	-5.81499103
TRIP10	-2.05792868	2.57510381	-1.54690285	0.17158314	0.99649919	-5.04655988
COBLL1	-2.06850999	3.502272	-0.37761921	0.71838526	0.99649919	-6.12675215
MPZL3	-2.06981849	3.23122487	-1.62649628	0.15369161	0.99649919	-5.06790488
XCL1	-2.07556195	4.97475794	-0.97250897	0.36742812	0.99649919	-6.07088435
CD82	-2.1006782	4.41371729	-0.90569735	0.39913945	0.99649919	-6.10249779
CD27	-2.10150321	1.67862489	-0.41379179	0.69305046	0.99649919	-5.47497693
TCF7	-2.11328701	7.55184218	-1.64069413	0.15069233	0.99649919	-5.44685774
MZB1	-2.12241236	1.22853298	-0.38955572	0.70997983	0.99649919	-5.50048903
TNFSF9	-2.12350065	3.60103952	-1.14595728	0.29437807	0.99649919	-5.70532046
BCAR3	-2.12788232	1.2953086	-0.67410505	0.52472456	0.99649919	-5.59683841
STAP1	-2.13248475	3.42859533	-0.97750506	0.36513789	0.99649919	-5.77151992
ADAM19	-2.13333889	4.14803527	-0.46569136	0.65745017	0.99649919	-6.15714595
NAV2	-2.14364605	1.34165793	-0.98992016	0.35949531	0.99649919	-5.12730431
XCL2	-2.14816968	5.09963263	-1.12407454	0.30287444	0.99649919	-5.85124442
HOXA5	-2.15224789	1.56613296	-1.16272597	0.28800394	0.99649919	-5.56264634
CXCR3	-2.16800522	5.13392062	-1.63602591	0.15167227	0.99649919	-5.38492835
CPNE5	-2.16850882	1.38532274	-0.28353799	0.78605503	0.99649919	-5.38531677
NBEA	-2.16855714	1.93184211	-0.49998239	0.63444664	0.99649919	-5.75529106
LEF1	-2.17360819	3.80299814	-0.98641236	0.36108257	0.99649919	-5.7641846
PTK2	-2.1739226	4.22173607	-0.95494516	0.37556878	0.99649919	-6.0271005
GEM	-2.17666794	1.05108507	-2.00664637	0.09035108	0.99649919	-4.27012971
LARGE1	-2.21308307	3.74684132	-1.22018338	0.26704624	0.99649919	-5.50509554
FHL1	-2.25287215	2.72078723	-1.07169296	0.32404509	0.99649919	-5.5792966

Gene	logFC	AveExpr	t	P.Value	adj.P.Val	B
CNKSR2	-2.2810257	1.52494552	-0.45468655	0.66492177	0.99649919	-5.56822352
CCDC141	-2.28153775	4.42189898	-0.97718944	0.36528223	0.99649919	-5.84604247
TTN	-2.28913893	8.54793053	-1.33115713	0.23029099	0.99649919	-5.83231393
BACH2	-2.31519476	5.87576191	-1.51428393	0.17946745	0.99649919	-5.46535146
ADCY3	-2.3207869	4.43928318	-1.18756302	0.27877817	0.99649919	-5.77739454
PHEX	-2.32180223	1.36239466	-0.65129189	0.53838592	0.99649919	-5.53920572
DUSP4	-2.32582662	3.13856876	-0.98870071	0.36004648	0.99649919	-5.98781047
ZC3H12B	-2.33604339	1.0037959	-1.89039488	0.10633459	0.99649919	-4.22045373
AQP3	-2.36452727	2.42129008	-1.23890003	0.26050907	0.99649919	-5.40611514
PPP1R9A	-2.36502538	4.65433259	-1.28234934	0.24586843	0.99649919	-5.61033213
PCDH9	-2.36798752	2.47779828	-0.53481129	0.61152898	0.99649919	-5.85374138
RIC3	-2.36986642	1.29281089	-0.67956857	0.52148617	0.99649919	-5.34196043
CNR2	-2.37514469	3.203277	-0.85581944	0.42413609	0.99649919	-5.73661079
LRP5	-2.40281508	1.62820474	-0.48078612	0.64727161	0.99649919	-5.61202063
NFIX	-2.41542214	3.30313443	-1.17322256	0.28407376	0.99649919	-5.60918337
STYK1	-2.42008022	1.09632921	-0.51813777	0.62244285	0.99649919	-5.30551686
ZEB1	-2.49842617	5.60122606	-1.73318919	0.13248463	0.99649919	-5.20952698
BAIAP3	-2.53005505	5.05474903	-1.58596447	0.16256967	0.99649919	-5.29516032
MCC	-2.56364788	1.36744634	-1.33682102	0.22854141	0.99649919	-4.81173269
COL4A3	-2.57249332	1.48888028	-0.37901565	0.71739966	0.99649919	-5.47725243
TTC24	-2.57763351	1.64048379	-1.25583771	0.25471355	0.99649919	-4.89142608
COL1A1	-2.60785547	1.2086694	-1.46550122	0.1918906	0.99649919	-5.08075446
LARGE2	-2.62080506	1.28820368	-0.33832684	0.74635297	0.99649919	-5.30950125
RUNX2	-2.66929394	4.67136446	-1.17605152	0.28302236	0.99649919	-5.81863643
IGLC3	-2.6941364	1.51121414	-0.29365621	0.77866565	0.99649919	-5.21525956
BTBD6P1	-2.72153572	2.02577173	-0.60846419	0.56463102	0.99649919	-5.38890717
MYO7A	-2.72269525	1.21988689	-0.44813639	0.66938899	0.99649919	-5.45254735
MICAL3	-2.74380032	6.32049898	-1.76853366	0.12610523	0.99649919	-5.17533155
PAWR	-2.836449	1.91791235	-0.63106605	0.55068409	0.99649919	-5.53496475
DERL3	-3.02929323	2.01182221	-0.62380922	0.55513874	0.99649919	-5.63218494
CCR7	-3.0321599	3.35824642	-0.58073071	0.58203363	0.99649919	-5.89114451
ITGA1	-3.10547492	1.73954671	-1.18550894	0.27953149	0.99649919	-4.90781971
SERPINE1	-3.17338112	2.46514764	-0.95936839	0.37350554	0.99649919	-5.7220421
KIF26B	-3.21536819	1.06855094	-0.65643409	0.53528704	0.99649919	-5.470863
SLC41A2	-3.27342381	1.23286908	-0.91807911	0.39310976	0.99649919	-5.19699942
CEP170B	-3.32393825	1.53317077	-0.81666359	0.44455331	0.99649919	-5.27512079
ITM2C	-3.41044756	2.97232072	-0.54296639	0.60623	0.99649919	-5.86374745
NELL2	-3.41129093	2.86618094	-0.50661704	0.63004568	0.99649919	-5.57130939
KIT	-3.41446184	2.54434807	-0.62967923	0.55153368	0.99649919	-5.5292713
COL4A4	-3.45174906	1.87902254	-0.47836779	0.64889682	0.99649919	-5.3113263
STARD10	-3.49113884	3.13029053	-1.68320019	0.14204424	0.99649919	-4.9056947
GPR183	-3.51937831	5.03773808	-1.16873303	0.28574914	0.99649919	-5.81382964
PTPRS	-3.7184834	3.17248252	-0.74846122	0.48177605	0.99649919	-5.93679561
DLGAP3	-3.72561177	1.18547324	-0.99549569	0.35698377	0.99649919	-4.87614325
AXIN2	-3.82385051	1.21200841	-0.42660539	0.68417652	0.99649919	-5.15580954
DPP4	-3.88598105	1.27231053	-0.57639483	0.58478274	0.99649919	-5.28830572
INPP4B	-3.92296182	2.03528694	-0.43770086	0.67653653	0.99649919	-5.30907193

Gene	logFC	AveExpr	t	P.Value	adj.P.Val	B
GZMK	-4.00279107	4.2401577	-1.08597145	0.31815673	0.99649919	-5.5978151
TMEM163	-4.03743835	1.10975509	-1.79942487	0.12077579	0.99649919	-4.21885036
DTX1	-4.08221201	3.2813977	-1.35810934	0.22207134	0.99649919	-5.25911641
HAPLN3	-4.13878232	3.3343926	-1.15087719	0.29249568	0.99649919	-5.50264528
IGFBP4	-4.1659336	1.84908834	-0.54781486	0.60309187	0.99649919	-5.39870241
IL7R	-4.19561835	5.51816664	-1.04413269	0.33566271	0.99649919	-5.70539847
ANO9	-4.33183487	3.24982344	-1.01868891	0.34668535	0.99649919	-5.36361324
CELSR1	-4.48448594	4.5975704	-1.26003575	0.25329464	0.99649919	-5.3868198
LSR	-4.54030645	3.38761047	-0.82341789	0.4409816	0.99649919	-5.41469231
TNFRSF11A	-4.72430524	3.63856035	-1.19701313	0.27533477	0.99649919	-5.36407193
PACSIN1	-5.24092203	1.8934766	-0.57300283	0.58693868	0.99649919	-5.31522519
